# Intronic Determinants Coordinate *Charme* lncRNA Nuclear Activity through the Interaction with MATR3 and PTBP1

**DOI:** 10.1016/j.celrep.2020.108548

**Published:** 2020-12-22

**Authors:** Fabio Desideri, Andrea Cipriano, Silvia Petrezselyova, Giulia Buonaiuto, Tiziana Santini, Petr Kasparek, Jan Prochazka, Giacomo Janson, Alessandro Paiardini, Alessandro Calicchio, Alessio Colantoni, Radislav Sedlacek, Irene Bozzoni, Monica Ballarino

**Affiliations:** 1 Department of Biology and Biotechnology “Charles Darwin,” Sapienza University of Rome, P.le A. Moro 5, 00185 Rome, Italy; 2 Czech Centre of Phenogenomics and Laboratory of Transgenic Models of Diseases, Institute of Molecular Genetics of the Czech Academy of Sciences, v.v.i., Prumyslova 595, 252 50 Vestec, Czech Republic; 3 Center for Life Nano Science@Sapienza, Istituto Italiano di Tecnologia, Viale Regina Elena 291, 00161 Rome, Italy; 4 Department of Biochemical Sciences “A. Rossi Fanelli,” Sapienza University of Rome, P.le A. Moro 5, 00185 Rome, Italy

**Keywords:** lncRNA, chromatin, epigenetic control, nuclear aggregates, ribonucleoparticle, introns, alternative splicing, myogenesis, muscle, CRISPR Cas9

## Abstract

Chromatin architect of muscle expression (*Charme*) is a muscle-restricted long noncoding RNA (lncRNA) that plays an important role in myogenesis. Earlier evidence indicates that the nuclear *Charme* isoform, named *pCharme*, acts on the chromatin by assisting the formation of chromatin domains where myogenic transcription occurs. By combining RNA antisense purification (RAP) with mass spectrometry and loss-of-function analyses, we have now identified the proteins that assist these chromatin activities. These proteins—which include a sub-set of splicing regulators, principally PTBP1 and the multifunctional RNA/DNA binding protein MATR3—bind to sequences located within the alternatively spliced intron-1 to form nuclear aggregates. Consistent with the functional importance of *pCharme* interactome *in vivo*, a targeted deletion of the intron-1 by a CRISPR-Cas9 approach in mouse causes the release of *pCharme* from the chromatin and results in cardiac defects similar to what was observed upon knockout of the full-length transcript.

## Highlights

• *pCharme* is the chromatin-retained isoform of the muscle-specific *Charme* lncRNA
• Intronic signals coordinate the association of *pCharme* with MATR3 and PTBP1
• The particle assembly prompts *pCharme* intron-1 chromatin retention
• Deletion of the intron-1 by CRISPR-Cas9 leads to heart defects in mouse

## Graphical Abstract

**Figure:**
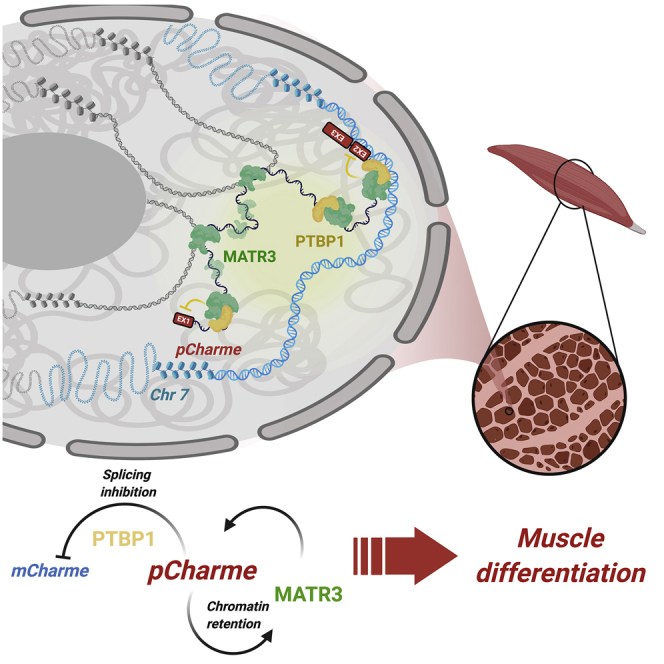

## Teaser

Desideri et al. study the role of the chromatin-retained isoform of *Charme*, a lncRNA that regulates myogenesis. They find that a MATR3/PTBP1/*pCharme* assembly drives the retention and the function of the lncRNA. CRISPR-Cas9 deletion of *pCharme* intron-1 leads to the transcript delocalization and to heart defects *in vivo*.

## Introduction

The discovery and characterization of functional long noncoding RNAs (lncRNAs) updated the notion that proteins are the unique determinants for cellular phenotypes, revealing the requirement of these transcripts in cell growth and differentiation, apoptosis, organ development, and function (Fatica and Bozzoni, 2014; Kopp and Mendell, 2018). Consistent with their crucial cellular functions, the dysregulation of lncRNA expression was found to be associated with multiple diseases, including cancer, neurodegeneration, and muscle disorders (Batista and Chang, 2013; Schmitz et al., 2016). In myogenesis, several archetypes of lncRNAs have been described that contribute to muscle physiology and related disorders through a wide range of molecular mechanisms (Martone et al., 2020). A significant portion of them was found to be functional in the nucleus, where they participate in cell-type-specific gene expression programs by influencing the epigenetic status, the function of the transcription factors, or the 3D architecture of chromatin domains (Engreitz et al., 2016). All these activities are temporally and spatially regulated by lncRNAs through their interaction with protein and nucleic acid moieties. Biochemical high-throughput approaches revealed that lncRNAs may serve as protein scaffolds, structuring ribonucleoprotein (RNP) aggregates and bringing proteins in proximity (Ribeiro et al., 2018). Their conformational versatility is unique and further amplified by splicing regulation, which leads to a variety of RNA structures by joining alternative combinations of sequences.

A further aspect regards the mechanisms that determine the nuclear or cytoplasmic localization of lncRNAs (Chen, 2016). Several lines of evidence suggest that nuclear export is the default pathway and that, in the absence of retention signals, lncRNAs are efficiently exported into cytoplasm (Miyagawa et al., 2012). Nevertheless, both the presence of *cis*-acting RNA motifs (Gudenas and Wang, 2018; Lubelsky and Ulitsky, 2018; Sunwoo et al., 2017) and the interaction with *trans*-acting regulators were shown to play an active role in lncRNA nuclear localization (Chin and Lécuyer, 2017; Guo et al., 2020; Palazzo and Lee, 2018). Introns have also been proposed as a mean to poise lncRNAs on the chromatin (Chorev and Carmel, 2012; Zuckerman and Ulitsky, 2019). However, how they influence the loading of specific proteins and how this contributes to the final distribution of lncRNAs in the nucleus are less known (Yu et al., 2015).

In mouse, we have recently identified and functionally characterized *Charme* (Chromatin architect of muscle expression) (Ballarino et al., 2015, 2018), a muscle-restricted lncRNA conserved in human that shapes myogenesis through the regulation of myoblast fusion and contraction genes. Consistently, *Charme* knockout mice show reduced lifespan as a consequence of muscle hyperplasia and a pronounced phenotype of cardiac remodeling (Ballarino et al., 2018). In skeletal and cardiac differentiating muscles, the alternative splicing (AS) of *Charme* primary transcript produces two main isoforms that acquire distinct subcellular distributions. On the chromatin, the unique isoform detected is *pCharme*, an 11-kb-long unspliced transcript harboring a very large intron-1, embedded between the first two exons. *pCharme* is the functional isoform that contributes to early myogenesis by controlling the 3D proximity of myogenic domains. Completion of intron-1 splicing leads to the production of a second isoform, *mCharme*, which escapes chromatin retention and translocates to cytoplasm. *In vitro* evidence suggests that this fully spliced transcript is not functional in myogenesis, as it fails to rescue the ability of *Charme*-ablated myoblasts to differentiate into myotubes (Ballarino et al., 2018). Thus, the presence of the intron-1 appears to be a distinctive determinant of *pCharme* muscular activity, although its functional significance has not been established yet. By using high-throughput biochemical approaches we found that intron-1 acts as a hub for the chromatin assembly of a specific RNP particle. Major constituents of such RNP are the RNA/DNA binding protein Matrin 3 (MATR3), a nuclear matrix component regulating chromatin structure and RNA transcription and processing (Banerjee et al., 2017; Coelho et al., 2016b), and the Polypyrimidine Tract Binding Protein 1 (PTBP1; also termed PTB and heterogeneous nuclear RNP I [hnRNP I]), a heterogeneous nuclear factor implicated in many steps of gene expression, including the regulation of AS (Hall et al., 2013; Robinson and Smith, 2006; Shen et al., 2004; Wagner and Garcia-Blanco, 2001). Interestingly, MATR3 was found as major interactor of PTBP1 in nuclear extracts, and both proteins were shown to co-regulate some AS events (Coelho et al., 2015, 2016a). Thus, the study of MATR3/PTBP1 overlapping networks raises particular interest, in light of the relevance of splicing regulation in biological transitions such as cellular differentiation and myogenesis (Bland et al., 2010; Castle et al., 2008). Using a RNAi-based knockdown approach in combination with gene expression analyses, we found that, in myotubes, PTBP1 acts as a repressor of intron-1 splicing. The concomitant interaction of the retained sequences with MATR3 reciprocally influences both MATR3 and *pCharme* performances. On one hand, MATR3 stabilizes the maintenance of *pCharme* on its chromatin locus; on the other hand, *pCharme* influences MATR3 chromatin binding. Indeed, chromatin immunoprecipitation sequencing (ChIP-seq) analyses performed in differentiated myotubes show a consistent decrease of MATR3 chromatin deposition upon *pCharme* depletion. In line with the functional role of intron-1, its deletion *in vivo* leads to cardiac dysfunction that mimics the cardiac phenotype observed in conditions of full-length *Charme* ablation (Ballarino et al., 2018). Overall, our data fill an important gap in the comprehension of the mechanism through which *Charme* contributes to myogenesis. We propose the existence of a circuitry in which the interaction between MATR3/PTBP1 and *pCharme* prompts intron-1 retention and, consequently, the chromatin maintenance and function of this lncRNA.

## Results

### *pCharme* Associates in the Nucleus with a MATR3/PTBP1-Containing Ribonucleoparticle

In muscles, AS generates two distinct isoforms of *Charme*, either with (*pCharme*) or without (*mCharme*) the 11-kb-sized intron-1. Intron-1 has a number of features that make it peculiar: (1) the high correlation between its retention and the maintenance of *pCharme* at the sites of transcription; (2) its evolutionary (human versus mouse) conservation, with a level of sequence identity (∼45%) very similar to the one displayed by the exonic sequences; (3) its contribution to myogenesis, as shown by the evidence that *mCharme* is unable to rescue the aberrant phenotype caused by *pCharme* and *mCharme* depletion in C_2_C_12_ myotubes (Ballarino et al., 2018). Collectively, these observations suggest a potential need of intron-1 for *pCharme* activity.

**Figure 1:**
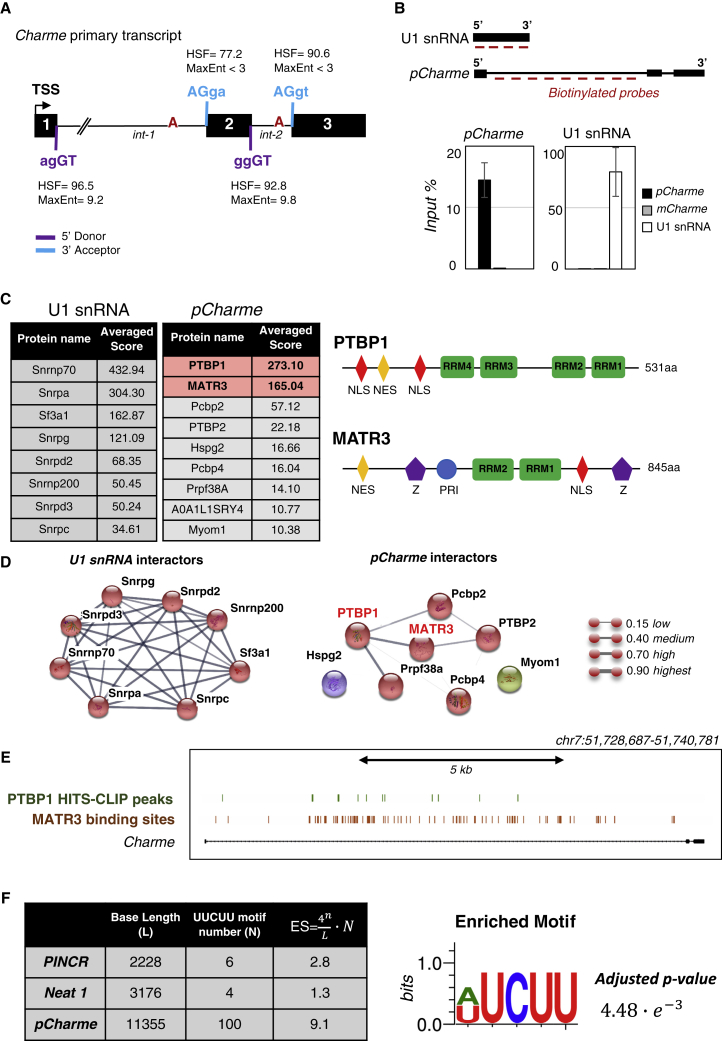
Identification of *pCharme* Protein Interactors (A) *In silico* analyses performed with the Human Splicing Finder (HSF) v.3.1 tool (Desmet et al., 2009) to identify splicing motifs within the *Charme* primary transcript. Consensus values span from 0 to 100 for HSF (threshold = 65) and −20 to +20 for MaxEnt (threshold = 3). Every signal with a score above the threshold is considered as a donor (violet) or acceptor (light blue) splice site. HSF scores above 80 are associated with strong splice sites. Branch point nucleotides are highlighted in red. TSS, transcription start site. (B) Upper panel: schematic representation of the antisense probes used for *pCharme* and U1 snRNA pulldown. Lower panel: quantification by qRT-PCR of *pCharme*, *mCharme*, and U1 transcripts in the *pCharme* and U1 snRNA pull-down samples. Values represent the percentage of RNA enrichment in respect to input. See Table S1 for probe and primer details. (C) Left: list of the top eight U1 snRNA and top nine *pCharme* interactors, as identified by MS. Proteins are ranked by the averaged protein score (full list is reported in Table S2). Right: schematic representation of MATR3 and PTBP1 protein domains. NLS, nuclear localization signal; NES, nuclear export signal; RRM, RNA recognition motif; Z, DNA binding C_2_H_2_ ZF domain; PRI, PTBP1-RRM interaction motif. (D) STRING functional network analysis (Szklarczyk et al., 2019) performed on U1 snRNA (left) and *pCharme* (right) protein interactors, as identified by MS analysis. Line thickness between nodes indicates the score for each functional connection between proteins. The combined score is computed by combining the probabilities from the different evidence channels (Neighborhood in the Genome, Gene Fusions, Co-occurrence Across Genomes, Co-Expression, Experimental/Biochemical Data, Association in Curated Databases) and corrected for the probability of random interactions. Minimum required interaction score: 0.15. The colors of the nodes represent the different functional clusters calculated using the MCL clustering methods (inflation parameter = 3). See Table S3 for details. (E) Visualization of PTBP1 (green) HITS-CLIP peaks and MATR3 (red) predicted binding sites along *pCharme* intron-1 genomic sequence. The murine genomic coordinates of the *Charme* locus are indicated (NCBI37/mm9). (F) Table shows the *in silico* search for UUCUU motifs in Neat1, PINCR, and *pCharme* nucleotide sequences. For each transcript, the base length (L), the motif number (N), and the UUCUU enrichment score (ES) are reported. ES was calculated according to the reported equation (Chaudhary et al., 2017). Statistical significance was evaluated using the AME software by comparing the (A/U)UCUU enrichment on *pCharme* intron-1 with a control set of intronic sequences with similar length.

To get insights into the mechanisms regulating intron-1 splicing, an *in silico* analysis of splicing-related *cis*-acting sequences was performed using the Human Splicing Finder (HSF) tool (Desmet et al., 2009). Indeed, many studies have found that common sequence features can predispose to intron retention, including the occurrence of weaker splice motifs in the retained introns compared to the constitutive ones. The examination of *Charme* primary transcript revealed that both intron-1 and intron-2 are flanked by 5′ and 3′ canonical splice sites. Moreover, a consensus branch point motif, followed by a polypyrimidine stretch, is located inside intron-1 ∼60 nt upstream of the intron-1/exon-2 boundary (Figures 1A, S1A, and S1B). The presence of canonical *cis*-acting splice sites and the constitutive intron-2 removal suggest that the retention of intron-1 and the accumulation of *pCharme* can be regulated by other inputs, including the intervention of specific RNA binding proteins acting as splice regulators. An UV crosslinked-based RNA affinity purification (RAP) approach (McHugh et al., 2015) was then applied in order to identify the nuclear interactors of *pCharme*. To this end, nuclear extracts from C_2_C_12_ myotubes were used as inputs and incubated with biotinylated probes antisense to intron-1 sequences (Figure 1B; Table S1). In parallel, pull-down efficiency was assayed with probes against U1 small nuclear RNA (snRNA), whose protein interactors are well characterized (McHugh et al., 2015). Quantitative RT-PCR (qRT-PCR) analyses performed on the precipitated RNAs confirmed the specific enrichment of *pCharme* and U1 snRNA in their respective samples (Figure 1B). To note, no specific enrichment was found for the cytoplasmic *mCharme* transcript in both samples, thus confirming the specificity of the pull-down for the *pCharme* isoform. Specific *pCharme* and U1 snRNA co-precipitated proteins were then identified by mass-spectrometry (MS) analyses (Table S2). As expected, many proteins found in the U1 precipitates were already known to be physically and functionally connected with U1 snRNA (McHugh et al., 2015). Notably, nine proteins (mean score > 10) were specifically enriched in *pCharme* samples compared to U1 (Figure 1C; Table S2), and a sub-group of them (e.g., MATR3, PTBP1, PTBP2, PCBP2, and PRPF38A) also showed a significant functional connection (Coelho et al., 2015; Wagner and Garcia-Blanco, 2001) (Figure 1D; Table S3). Interestingly, Gene Ontology (GO) term enrichment analysis performed on the top eight *pCharme* protein interactors revealed a significant enrichment of RNA binding (GO: 0003723) and splicing regulation (GO: 0033119) terms within the molecular function and biological process categories, respectively (Figure S1C).

Among the interactors, PTBP1 and MATR3 were found as the two uppermost *pCharme* partners (score > 100) (Figure 1C, left panel). PTBP1 is a well-known splicing regulator containing four closely related RNA recognition motifs (RRMs; Figure 1C, right panel) and contributing to the formation of the splicing machinery by cooperation with PTBP2 and PCBP2 (Wagner and Garcia-Blanco, 2001). The other interactor, MATR3, is highly conserved and one of the most abundant proteins of the nuclear matrix. It has been linked to a variety of functions and shown to regulate the nuclear organization due to its ability to bind both DNA via the Z-DNA binding domain and RNA via the RRM1 and RRM2 motifs (Figure 1C, right panel) (Uemura et al., 2017). Interestingly, MATR3 was also shown to interact with PTBP1 and with other proteins involved in splicing (Coelho et al., 2015) or nuclear dynamics regulation (Coelho et al., 2016a) via the seven-amino-acid PTB-RRM2 Interactive (PRI) motif (Figure 1C, right panel). PTBP1 and MATR3 were also shown to actively bind different RNA templates, including lncRNAs, to synergistically regulate a variety of nuclear processes (Cerase et al., 2019; Coelho et al., 2015; Pandya-Jones et al., 2020).

As a further validation, the direct interaction of these two factors with *pCharme* RNA was confirmed by MATR3-crosslinking immunoprecipitation (CLIP) (Figure S1D) and high-throughput sequencing of RNA isolated by PTBP1-crosslinking immunoprecipitation (HITS-CLIP) experiments (Masuda et al., 2012; Yang et al., 2015) (Figure 1E). MATR3-CLIP assay revealed a unique enrichment of *pCharme* but not *mCharme*, supporting a direct and specific binding of MATR3 with the nuclear isoform (Figure S1D). The interaction observed by biochemical assays was further corroborated by a sequence-based binding prediction analysis that revealed the presence of ∼100 MATR3 CU-rich consensus-binding motifs (Coelho et al., 2015) within intron-1 (Figures 1E and 1F). These sites were found statistically enriched in *pCharme* RNA as compared to introns of comparable length of the C_2_C_12_ transcriptome (Figure 1F, right panel), and more than three (enrichment score [ES], 9.1 versus 2.8) and seven (ES, 9.1 versus 1.3) times enriched in respect to PINCR and Neat1 lncRNAs, previously shown to bind MATR3 (Banerjee et al., 2017; Chaudhary et al., 2017) (Figure 1F, left panel). By surveying PTBP1 HITS-CLIP data (Yang et al., 2015), a total of 11 PTBP1 binding sites were also identified within the intron-1, the majority of them (91%) overlapping the 69% of MATR3 binding sites (Figure 1E).

**Figure 2:**
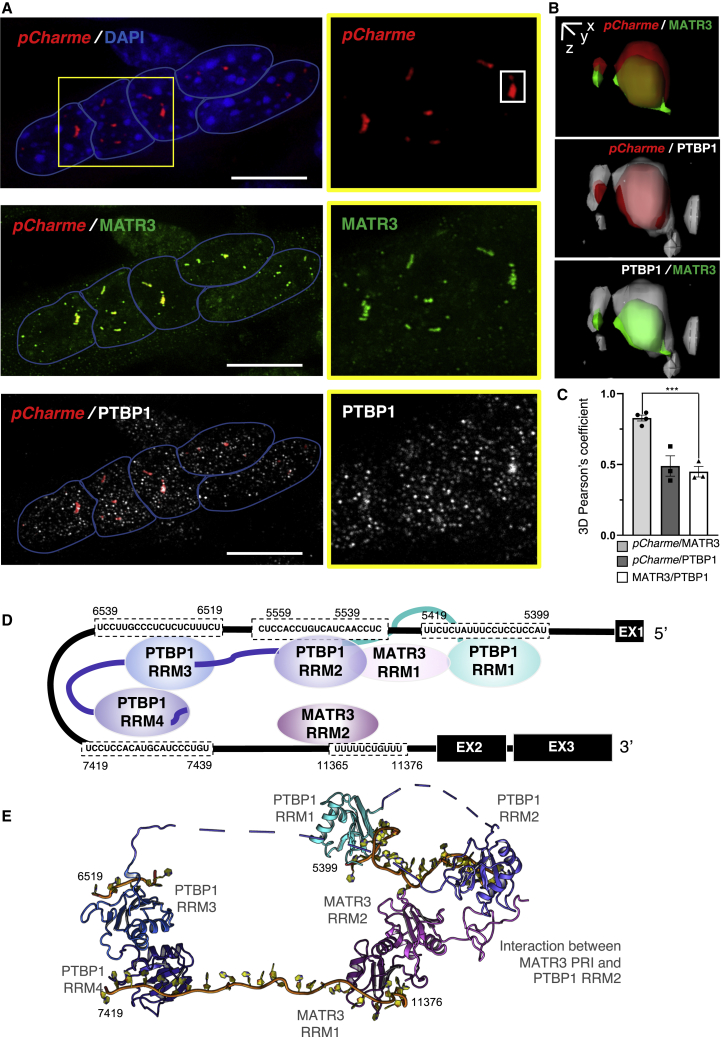
Study of the RNA/Protein Interactions between *pCharme* and PTBP1/MATR3 (A) *pCharme* RNA-FISH (red, upper panel) and co-staining *pCharme* (red)/MATR3 (green, middle panel) or *pCharme* (red)/PTBP1 (white, bottom panel) in differentiated C_2_C_12_ myotubes. Blue lines indicate the edge of the nucleus. DAPI, 4′,6-diamidino-2-phenylindole. Magnification of the yellow box is shown on the right. Scale bars, 15 μm. (B) Selected 3D-rendered iso-surface views showing MATR3/PTBP1 occupancy on *pCharme* transcript. (C) Scatter dot blot representing the quantification of *pCharme*/MATR3/PTBP1 overlapping signals. 3D-Pearson’s correlation coefficient indicates the means ± SEM calculated from images shown in (A) from four (*pCharme*/MATR3) and three (*pCharme*/PTBP1 and MATR3/PTBP1) biological replicates. ^∗∗∗^p < 0.001, unpaired Student’s t test. (D) Neighboring MATR3 and PTBP1 binding sites (MATR3 consensus sequence [CT][CT]TTTCT.TTT, as reported in Uemura et al. [2017] and PTBP1-CLIP data and consensus sequence T[CT]T[CT][CT], as reported in Xue et al. [2009]) were mapped on *pCharme* intron-1 (black line). RRM, RNA recognition motif. (E) Hypothetical RNP complex comprising MATR3, PTBP1, and *pCharme* assembled on intron-1 (orange line), based on the structural data available in the Protein Data Bank. RRM, RNA recognition motif; PRI, PTBP1 RRM interaction motif.

The physical proximity between PTBP1, MATR3, and *pCharme* in myotubes was further analyzed by a combined immunofluorescence (IF) and RNA-fluorescence *in situ* hybridization (FISH) approach (Figures 2A and 2B). Quantitative analysis of the overlapping signals by 3D Pearson’s correlation coefficient highlighted the formation of MATR3/*pCharme*, PTBP1/*pCharme*, and MATR3/PTBP1 nuclear aggregates (Figure 2B). Notably, the MATR3/*pCharme* colocalization strength was 2-fold higher than for MATR3/PTBP1 (Figure 2C), a well-known MATR3 protein interactor (Coelho et al., 2016a; Coelho et al., 2015). By analyzing the nuclear fluorescence, we found that 78% of *pCharme* signals overlap with MATR3 and that 20% of MATR3 signals colocalize with *pCharme* (Figure S2). As RRMs are required for RNA binding, we focused on four RRM motifs of PTBP1 (regions 5,399–5,419, 5,539–5,559, 6,519–6,539, and 7,419–7,439) and on a RRM2 motif of MATR3 (region 11,365–11,376) to model the stereometric interaction of the two proteins with intron-1. This led to the assembly on intron-1 of a hypothetical RNP complex formed by MATR3, PTBP1, and *pCharme* (Figures 2D and 2E), which corroborates the *in vitro* observations and supports the role of intron-1 as a scaffolding platform for the assembly of proteins potentially involved in *pCharme* biogenesis and function.

### Functional Interplay between *pCharme* and the PTBP1/MATR3-Containing Ribonucleoparticle

**Figure 3:**
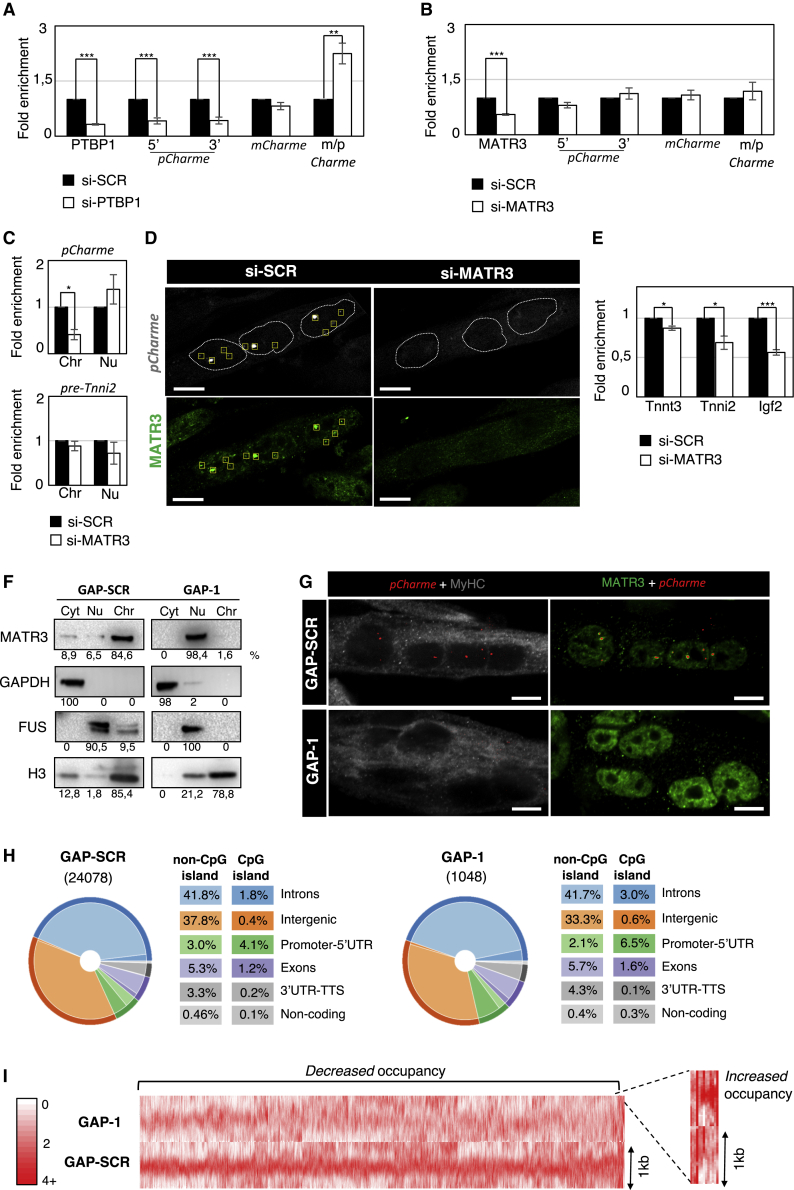
Functional Analysis of the *pCharme*-PTBP1/MATR3 Interaction in Myotubes (A) qRT-PCR quantification of PTBP1, *pCharme* (amplified at both 5′ and 3′ intron-1 ends), and *mCharme* levels in 2-day differentiated myotubes treated with si-SCR or si-PTBP1. Data were normalized to GAPDH (Glyceraldehyde 3-phosphate dehydrogenase) mRNA and represent means ± SEM of five independent experiments. The *mCharme/pCharme* ratio was obtained by dividing *mCharme* by *pCharme* 5′ and *pCharme* 3′ averaged expression levels. (B) qRT-PCR quantification of MATR3, *pCharme* (amplified at both 5′ and 3′ intron-1 ends), and *mCharme* levels in 2-day differentiated myotubes treated with si-SCR or si-MATR3. Data were normalized to GAPDH mRNA and represent mean ± SEM of three independent experiments. The *mCharme/pCharme* ratio was obtained by dividing *mCharme* by *pCharme* 5′ and *pCharme* 3′ averaged expression levels. (C) qRT-PCR quantification of *pCharme* (upper panel) and Tnni2 precursor (pre-Tnni2) (lower panel) RNA levels in chromatin (chr) and nucleoplasmic (nu) fractions from 2-day differentiated myotubes treated with si-SCR or si-MATR3. Data were normalized to the GAPDH precursor (pre-GAPDH) RNA and represent means ± SEM of three independent experiments. (D) Representative single focal plane images from 2-day differentiated myotubes of combined RNA-FISH/IF (green) showing *pCharme* localization upon MATR3 ablation. *pCharme* RNA (gray) and MATR3 protein (green) signals are detected in normal (si-SCR) and MATR3-depleted (si-MATR3) cells. Yellow squares indicate *pCharme/*MATR3 colocalized signals. Dashed lines indicate the edge of the nuclei. Scale bars, 10 μm. (E) qRT-PCR quantification of Tnnt3, Tnni2, and Igf2 mRNA levels in 2-day differentiated myotubes treated with si-SCR or si-MATR3. Data were normalized to GAPDH mRNA and represent means ± SEM of three independent experiments. (F) Western blot analysis of MATR3 in cytoplasmic (cyt), nucleoplasmic (nu), and chromatin (chr) fractions from 2-day differentiated myotubes treated with GAP-SCR or GAP-1. The quality of fractionation was tested with GAPDH, FUS (fused in sarcoma), and histone H3 proteins. Quantification analyses of the chemiluminescent signal were performed with the ImageJ tool. The relative abundance of the different proteins in each specific compartment is indicated as percentage values. (G) Representative single focal plane images from 2-day differentiated myotubes of combined RNA-FISH/IF showing MATR3 localization upon *pCharme* ablation. *pCharme* RNA (red), MyHC (myosin heavy chain) protein (gray), and MATR3 protein (green) signals are detected in normal (GAP-SCR) and *pCharme*-depleted myotubes (GAP-1). Scale bars, 10 μm. (H) MATR3 genomic occupancy, as obtained by ChIP-seq analyses from GAP-SCR (left) and GAP-1 (right) samples. For each category, the percentage of MATR-3 occupancy is reported in the box legend. (I) Heatmap of MATR3 chromatin occupancy centered on the middle of peaks (±500 bp) differentially called between GAP-SCR and GAP1 conditions. Red, high read density; white, low read density. See Table S1 for details. ^∗^p < 0.05; ^∗∗∗^p < 0.001, unpaired Student’s t test.

To gain further insights into the newly identified interactions, we tested the impact of PTBP1 and MATR3 depletions on the accumulation of *pCharme* and *mCharme* transcripts. In differentiated myotubes, *mCharme* represents the fully spliced version of *pCharme*, and the two isoforms display the same RNA stability (Ballarino et al., 2018). To directly test whether PTBP1 controls the splicing of intron-1, we knocked down PTBP1 expression in differentiated myotubes and examined the changes in *mCharme* and *pCharme* relative abundances by qRT-PCR. In line with its role as splicing repressor, PTBP1 depletion led to a ∼2.2-fold increase in the *mCharme*/*pCharme* ratio due to enhanced splicing of intron-1 (Figure 3A). In contrast to PTBP1, MATR3 depletion did not produce any significant effect on *p*C*harme* and *mCharme* levels (Figure 3B). Since MATR3 has been described as nucleator of chromatin activities (Coelho et al., 2015, 2016b), we then hypothesized its requirement for *pCharme* chromatin localization. The initial visualization of MATR3 by IF and *pCharme* by RNA-FISH during a time course of C_2_C_12_ cell differentiation revealed an intriguing correlation between these two factors. Indeed, while in proliferating cells (growth medium; GM), where *pCharme* is not expressed, MATR3 signals appeared diffused, they became more punctate and discrete in concomitance with the emergence of *pCharme* nuclear spots in the 1 and 2 days (differentiated medium; DM1 and DM2) differentiated myotubes (Figure S3A). Starting from this observation, we quantified, by qRT-PCR, the distribution of *pCharme* between chromatin and nucleoplasmic fractions isolated from MATR3-depleted and control myotubes. This analysis revealed reduced levels of the chromatin-associated *pCharme* in MATR3-depleted cells, with a slight increase of the transcript in the nucleoplasmic fraction (Figure 3C, upper panel). Notably, the chromatin delocalization was not observed when the Troponin i2 precursor transcript (pre-Tnni2) was analyzed in parallel as a control (Figure 3C, lower panel). The influence of MATR3 levels on *pCharme* localization was further confirmed by RNA-FISH analyses showing a strong decrease of *pCharme* chromatin foci (Figure 3D) in MATR3-depleted myotubes (si-MATR3) compared with control (si-SCR) cells. Chromatin clearance correlates with dysregulated expression of transcripts (i.e., insulin-like growth factor 2 [Igf2], troponin t3 [Tnnt3], and troponin i2 [Tnni2]) (Figure 3E) previously identified as *pCharme* direct targets (Ballarino et al., 2018).

To study the possible two-way nature of this interaction, we then examined the MATR3 response to *pCharme* downregulation. Again, qRT-PCR and western blot analyses revealed no changes in MATR3 accumulation upon *pCharme* depletion, both at mRNA and protein steady-state levels (Figure S3B). However, consistent with a reciprocal role of *pCharme* in guiding MATR3 chromatin localization, western blot analysis performed on cytoplasm, nucleoplasm, and chromatin fractions showed a mis-localization of MATR3 from chromatin to nucleoplasm upon *pCharme* downregulation (Figure 3F). A similar effect was also found when MATR3 localization was examined by IF staining performed on control (GAP-SCR) and *pCharme* (GAP-1)-depleted myotubes (Figure 3G). Indeed, while, in control (GAP-SCR) myotubes, a distinct and punctate distribution of MATR3 was clearly visible, with the highest signal intensity found in correspondence to *pCharme* foci, MATR3 nuclear staining appeared more diffuse and intense in the nucleoplasmic compartment of *pCharme*-depleted cells (Figures 3G and S3C).

As MATR3 chromatin localization was found to be *pCharme* dependent, we then explored whether *pCharme* might affect MATR3 binding to specific genomic sites. To this purpose, we compared the MATR3 DNA binding profile in control (GAP-SCR) and *pCharme*-depleted (GAP-1) myotubes by MATR3 ChIP-seq analysis. In both the conditions, we found a broad distribution of the protein binding along the genome with a preference toward CpG islands and genic regions (Figures 3H and S3D). Despite the genomic regions bound by MATR3 displaying the same distribution in GAP-SCR and GAP-1-treated myotubes, a substantial reduction in the number of MATR3-bound regions was observed in the absence of *pCharme* (Figures 3H and S3D). Moreover, differential binding analysis of ChIP-seq peaks revealed that out of the 2,292 differentially enriched regions, the vast majority exhibit a reduced MATR3 occupancy upon *pCharme* knockdown (Figures 3I and S3E). Among the 12 genomic targets displaying increased MATR3 occupancy in *pCharme*-deficient cells (Figure 3I), we found the locus encoding for Neat1 (Figure S3E), a lncRNA that was already known to be associated with MATR3 in muscle cells (Banerjee et al., 2017). To note, genes in proximity of the differentially bound MATR3-contacted sites (±25 kb) are slightly but significantly enriched in *pCharme* targets (i.e., those genes whose expression level changes upon *Charme* depletion; chi-square with Yates correction test, p = 0.0344; Ballarino et al., 2018). These data correlate with the existence of a functional interplay between *pCharme* and MATR3, which acts in myoblasts to coordinate their respective chromatin localization and activities. Together with the evidence of PTBP1-mediated regulation of intron-1 retention, this network of interactions establishes the appropriate environment necessary for *pCharme* function.

### Mice with a Deletion of *pCharme* Intron-1 Develop Cardiac Dysfunction

**Figure 4:**
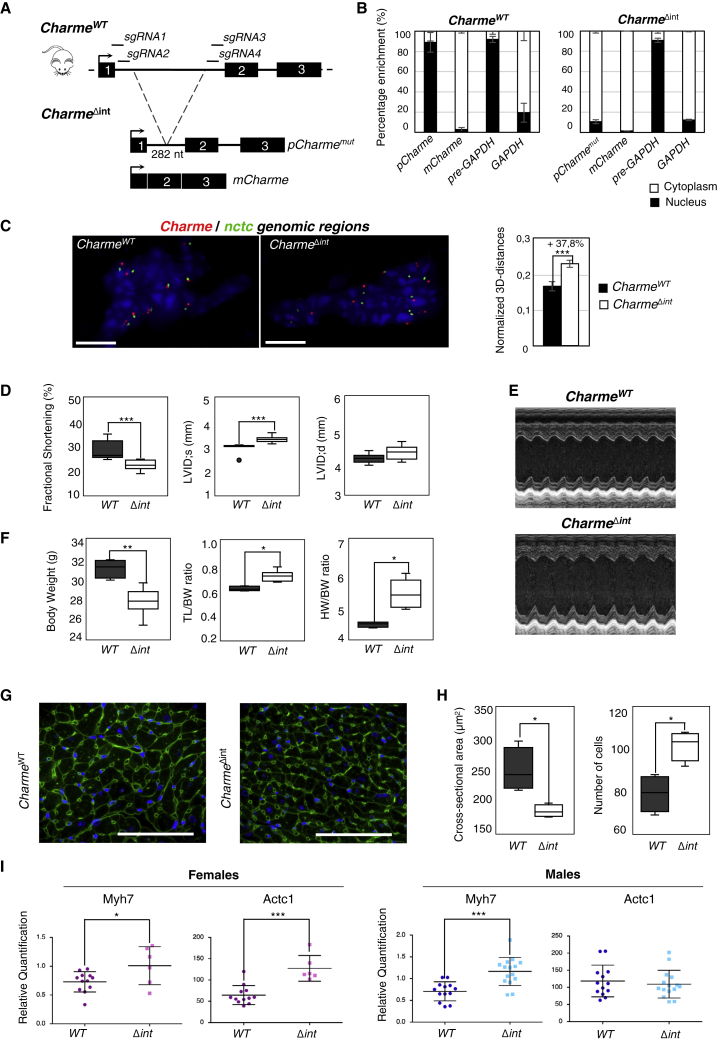
*pCharme* Intron-1 Deletion by CRISPR-Cas9 Leads to Cardiac Dysfunction *In Vivo* (A) Schematic representation of WT (Charme^WT^) and *Charme*-edited (*Charme*^Δint^) genomic loci. The positions of the single guide RNAs (sgRNAs) used in this study are indicated. The two isoforms (*pCharme*^*mut*^ and *mCharme*) produced by the edited locus are also indicated. (B) Percentage of subcellular enrichment of *pCharme*, *pCharme*^*mut*^, and *mCharme* in cytoplasmic and nuclear fractions performed through qRT-PCR from heart of *Charme*^WT^ (upper panel) and *Charme*^Δint^ (lower panel) 6-week-old mice. The quality of the fractionation was tested with mature (GAPDH) and precursor (pre-GAPDH) RNAs. (C) DNA/DNA FISH in adult (6-week-old) cardiac tissues. Left panel: representative DNA/DNA FISH 2D images for *Charme* (red signals) and *nctc* (green signals) genomic regions in *Charme*^*WT*^ and *Charme*^*Δint*^ on transverse frozen sections of cardiac tissues. Nuclei were highlighted by DAPI staining (blue signal). Scale bars, 10 μm. Right panel: quantification of the interallelic *Charme*/*nctc* 3D distances in *Charme*^+/+^ and *Charme*^*Δint*^ cardiac tissues. The 3D distances, measured on z stack confocal images, were normalized to nuclei diameter and represented as means ± SD of three biological replicates. The percentage increases of the mean values in respect to *Charme*^*WT*^ are indicated. (D) Echocardiographic measurement of left ventricular FS and internal dimensions at end-systole (LVID; s) and end-diastole (LVID; d) of 36-week-old *Charme*^WT^ (WT; dark gray box) and *Charme*^Δint^ (Δint; white box) female hearts. *Charme*^WT^, n = 7; *Charme*^Δint^, n = 13. (E) Representative short-axis M-mode echocardiographic images of 36-week-old female *Charme*^WT^ and *Charme*^Δint^ hearts. (F) Body weight, tibia length/body weight (TL/BW), and heart weight/body weight (HW/BW) ratios of 36-week-old WT (dark gray) and Δint (white) female mice. (G) Representative cardiac cross-sections stained by Alexa Fluor 488-conjugated wheat germ agglutinin (green) and DAPI (blue) of WT and *Charme*^Δint^ hearts. Scale bars, 100 μm. (H) Number of cardiomyocytes counted per visual field and average cross-sectional area of individual cardiomyocytes of 36-week-old female WT (dark gray box) and Δint (white box) hearts. n = 4. 80–120 of cardiomyocytes per individual sample were analyzed. (I) qRT-PCR quantification of Myh7 and Actc1 transcripts in WT (purple dots) and Δint (magenta dots) female (left) and in WT (blue dots) and Δint (cyan dots) male (right) heart tissue in 2-week-old mice. Data were normalized to HPRT (hypoxanthine-guanine phosphoribosyltransferase) mRNA. Female mice: WT, n = 12; Δint, n = 6. Male mice: WT, n = 13; Δint, n = 15. See Table S1 for details. ^∗^p < 0.05; ^∗∗^p < 0.01; ^∗∗∗^p < 0.001, unpaired Student’s t test.

Given the crucial role of intron-1 for *pCharme* localization and function *in vitro*, we applied a CRISPR-Cas9 gene editing approach to generate a mouse model (*Charme*^Δint^) carrying intron-1 deletion. *In silico* design and evaluation of Cas9 guide RNAs was performed to delete 90% of intron-1 sequences (Figures 4A, S4A, and S4B), which contained all the possible MATR3 and PTBP1 binding sites. Only 282 residual nucleotides of intron-1 were left in the genome of mutant mice (*Charme*^Δint^), with the purpose to maintain unaltered the splicing into *mCharme*. Since the major phenotype observed in our previous *Charme*^−/−^ mouse model was at the level of the heart (Ballarino et al., 2018), we focused our analysis on the cardiac muscle. RT-PCR analyses on the RNA isolated from wild-type (WT) and *Charme*^Δint^ muscles confirmed that a shorter *pCharme* version, with the expected size, was produced (*pCharme*^mut^) and that, in line with our *in silico* predictions (Figures 1A, S1A, and S1B), constitutive splicing of this transcript still occurred (Figure S4C).

In agreement with the importance of the entire intron-1 for *pCharme* localization, biochemical sub-cellular fractionation of cardiac tissues from 6-week-old mutant mice revealed strongly reduced *pCharme*^mut^ chromatin levels, with aberrant increase in the cytoplasmic compartment (Figure 4B). This led to a significant increase in the 3D spatial distance between the *Charme* gene and its main interacting locus *nctc*, as measured by DNA-DNA FISH (Figure 4C), and to a concomitant alteration of Igf2 expression (Figure S4D), formerly described as a direct *pCharme* target (Ballarino et al., 2018). To determine the impact of *pCharme*^*mut*^ mislocalization on *Charme*^Δint^ cardiac functions, a cohort of animals was followed, and echocardiography was performed at various ages. At 6 and 12 weeks of age, there were no differences between control and mutant hearts (data not shown). However, at 36 weeks of age, remarkable left ventricular dilatation and reduction in fractional shortening (FS) were detected in female *Charme*^Δint^ hearts in comparison with their WT female counterparts (Figures 4D and 4E), whereas no changes were observed in males (Figure S4E). Accordingly, body weights of all mutant mice were not statistically different from those of WT littermates throughout their lives (Figure S4F). Only in *Charme*^Δint^ females, at 36 weeks of age, a significant drop in their body weight was observed when compared with WT females (Figure 4F). The observed increase in heart/body weight ratio and tibia length/body weight ratio in *Charme*^Δint^ females was mainly due to the decrease in body weight (Figure 4F), as tibia length and heart weight did not differ (data not shown). At this age, animals were sacrificed for morphometric and histological analyses. Morphometric analysis of myofiber dimensions revealed a significant decrease (25.6%) in cross-sectional area and increase in their number (Figures 4G and 4H). Together, these findings indicate that the targeted intron-1 deletion is sufficient to cause dilated cardiomyopathy, which is manifested by reduced left ventricular function with greater ventricular dilatation and more pronounced wall thinning in *Charme*^Δint^ females. In *Charme*^Δint^ males, such a cardiomyopathy does not develop up to this age. However, at age of 1 year, a tendency (not statistically significant) in later onset of ventricular dysfunction due to dilatation was observed in *Charme*^Δint^ males as well (Figure S4E). An attractive difference between the two sexes also emerged when the expression of two fetal genes, Myosin Heavy Chain 7 (Myh7) and Alpha-Cardiac Actin (Actc1) (Cui et al., 2020), was analyzed from the hearts of 2-week-old mice. Since the re-activation of the fetal gene program is a hallmark of numerous heart failure conditions (Ames et al., 2013; Taegtmeyer et al., 2010), the evaluation of their expression in young adult animals was of particular interest within the framework of our work. While a significant upregulation of Myh7 was found in both *Charme*^Δint^ females and males (Figure 4I), an increased expression of Actc1 was detected only in *Charme*^Δint^ females compared to *Charme*^WT^ ones (Figure 4I). Even if still preliminary, these results pave the way for future investigations to ascertain whether an altered expression of a larger subset of genes than Actc1 may contribute to the outcome of gender-specific phenotypes. Thus, there is a need for future work on a more comprehensive profiling of *Charme*^*WT*^ and *Charme*^Δint^ cardiac transcriptomes.

Overall, the analyses of *Charme*^Δint^ hearts evidenced morphological alteration and functional dysfunction of the heart, thus confirming the relevance of *Charme* locus in the control of proper muscle differentiation and homeostasis. Moreover, the cytoplasmic delocalization of the mutant *pCharme* transcript and the existence of an interesting muscle phenotype *in vivo* corroborate the pivotal importance of intron-1 sequences for the retention of *pCharme* within a proper chromatin milieu, a compelling necessity for its architectural activity.

## Discussion

**Figure 5:**
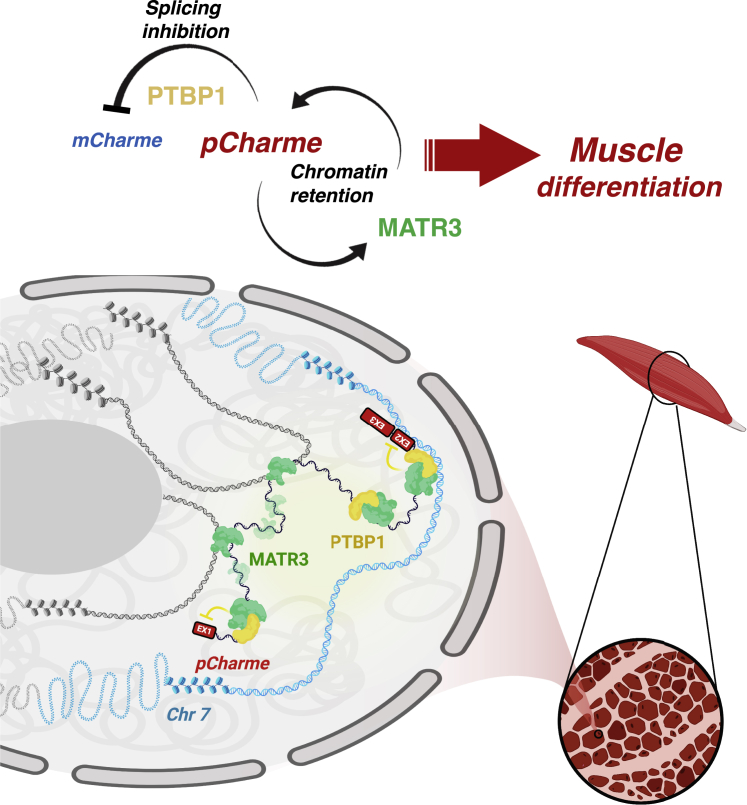
Proposed Model for the Functional Interplay between *pCharme* lncRNA and MATR3/PTBP1 Proteins In differentiating myotubes, MATR3 and PTBP1 are recruited on *Charme* locus through interactions mediated by the transcribed intron-1. Upon binding, PTBP1 acts as a splicing repressor, thus delaying the splicing of intron-1. Intron-1 acts as a hub for MATR3 binding, which culminates with (1) the overall stabilization of the *pCharme isoform* on the chromatin and (2) the coordination of MATR3 chromatin recognition. The model was created with BioRender.com.

Intron retention within mature RNA transcripts is expected to cause dramatic outcomes on the resulting proteins when it occurs inside coding sequences. As a consequence, cells have evolved several mechanisms of surveillance that rapidly degrade the aberrant transcripts and prevent them to undergo the next steps of RNA metabolism. Nevertheless, several advantages have also been ascribed to intron retention, especially in light of its contribution to increase vertebrate complexity (Schmitz et al., 2016). In this scenario, lncRNAs, which are devoid of any coding-sequence constraint, might be more prone to leverage the intron-retention scheme, thus expanding the repertoires of possible sequences with their related functions. Therefore, introns that have long been considered as junk material can be reinterpreted as drivers that amplify transcriptome diversity and contribute to shape lineage-specific identities (Jacob and Smith, 2017). In our study, we found that the retention of intron-1 contributes to the chromatin stabilization and activity of *pCharme*, a tissue-specific lncRNA previously identified as functional in myogenesis (Ballarino et al., 2018). Evidence to functionality was initially supported by the impressive conservation of intron-1 in mammals at the level of sequence identity (∼45%) and retention within the final *pCharme* transcript (Ballarino et al., 2018). Herein, the use of high-throughput biochemical approaches allowed the identification of MATR3 and PTBP1 as the predominant intron-1 protein interactors. Besides their myogenic potential, MATR3 and PTBP1 have been described in literature as nuclear-localized factors involved in many aspects of RNA processing (Coelho et al., 2015, 2016b; Wagner and Garcia-Blanco, 2001). Nevertheless, evidence for their possible function in *pCharme* metabolism has been missing. Consistent with a role as splicing repressor, in differentiating myotubes, PTBP1 downregulation leads to a peculiar increase of intron-1 splicing. Thus, in normal conditions, the binding of PTBP1 to *pCharme* speaks for a splicing-dependent mechanism that counteracts the production of *mCharme* and ensures the persistence of intron-1 to later stages of differentiation. The intronic retention confers to *pCharme* the ability to bind MATR3, which, in turn, stabilizes *pCharme* chromatin maintenance, as revealed by the strong delocalization of the lncRNA in MATR3-interfered myotubes. This mechanism resembles the chromatin-tethering activity recently ascribed to the U1snRNP, in which depletion alters the localization of a big portion of chromatin-retained lncRNAs (Yin et al., 2020). The intimate crosstalk between MATR3 and *pCharme* intron-1 turned out to have a genome-wide echo on the chromatin-recognition dynamics as *in vitro* depletion of the lncRNA impacts on MATR3 chromatin occupancy and culminates with its delocalization to nucleoplasm. The *pCharme*-dependent distribution of MATR3 in the nucleus seems to be broader than the *pCharme* localization to discrete chromatin foci. Even though the different sensitivities of the applied methodologies may have contributed to this apparent discrepancy, one could also interpret these data by assuming the presence of multiple MATR3 targets within the *pCharme* foci. In addition, MATR3 nuclear distribution might be also indirectly influenced by the myogenic impairment caused by *pCharme* depletion. In fact, in differentiating cells, MATR3 IF signals appear more diffuse in the nucleoplasm, with respect to mature myotubes. Overall, these results propose a model in which the lncRNA coordinates the crosstalk between MATR3 and its chromatin targets and vice versa (Figure 5).

The identification of MATR3 and PTBP1 as major *pCharme* interactors represents an important step forward into the characterization of the lncRNA mechanism of action. The ability of nuclear lncRNAs to bind one or more proteins makes them suitable platforms for the assembly of different ribonucleoparticles with roles in transcription and chromatin modification (Ribeiro et al., 2018). This scaffolding activity also serves for the binding of several hnRNPs that, in turn, lead to the tethering of lncRNAs to chromatin (Garland and Jensen, 2020). In this scenario, paradigmatic examples have been proposed to explain how lncRNAs control the homeostasis of different aggregates, such as speckles, paraspeckles, and X chromosome condensates (Chen et al., 2016; Clemson et al., 2009; Naganuma et al., 2012; Pandya-Jones et al., 2020; Tripathi et al., 2010). Notably, the binding of NEAT1 to MATR3 was proposed to regulate paraspeckle physiology and function in muscle cells (Banerjee et al., 2017). Thus, we speculate that the capacity of the protein to nucleate specific chromatin domains might be assisted by diverse classes of RNAs, depending on the cellular context. The regulation of MATR3 localization by *pCharme* is of particular interest in the context of myogenesis. Indeed, MATR3 mutations have been associated with familial amyotrophic lateral sclerosis (ALS) and myopathies, including cardiac developmental defects (Johnson et al., 2014; Müller et al., 2014; Quintero-Rivera et al., 2015; Senderek et al., 2009). Recently, it has been observed that the expression of MATR3 in spinal cord, cardiac, and skeletal muscle, which is the highest at the embryonal stages, decreases during postnatal mouse development to result at very low levels in adults (Quintero-Rivera et al., 2015; Rayaprolu et al., 2016). Since spinal cord and skeletal muscle are pathologically affected in ALS and distal myopathy, the low levels of MATR3 in adults could suggest that these two tissues are peculiarly susceptible to alterations in MATR3 function. According to these findings, Moloney and colleagues demonstrated that the overexpression of both the WT and the ALS-associated mutant MATR3 (MATR3^F115C^) in mouse muscles leads to the outbreak of a distinct phenotype correlated to muscle atrophy (Moloney et al., 2018). Moreover, it has also been reported that variations in MATR3 expression are responsible for cardiac defects in both mouse and human (Quintero-Rivera et al., 2015). Finally, the targeted deletion of the second RNA binding domain (RRM2) of MATR3 drives the formation of non-physiological, intranuclear, phase-separated, droplet-like structures (Gallego-Iradi et al., 2019; Iradi et al., 2018). Overall, these observations suggest that MATR3 abundance and behavior need to be tightly supervised to prevent the onset of pathological conditions. Due to their tissue specificity and structural versatility, lncRNAs represent suitable candidates to exert this function. Our *in vivo* studies on *Charme*^Δint^ mice suggest that *cis*-acting sequence elements constitute important determinants for *pCharme* function. Differently from the previous *Charme*^−/−^ mouse model, in which the insertion of a synthetic poly(A) cassette led to the ablation of all the *Charme* isoforms, the site-directed mutagenesis applied for the generation of the *Charme*^Δint^ animals resulted in the replacement of *pCharme* with the intron-1 deleted mutant, *pCharme*^mut^. In homozygous mice, this mutant transcript is not retained on the chromatin anymore, possibly due to the absence of all the MATR3 and PTBP1 predicted binding sites. Similar to what was observed in mice with the full-length *pCharme* transcript ablation (Ballarino et al., 2018), the partial intron-1 deletion led to the appearance of cardiac anomalies that involve an altered morphology of the ventricles, muscle hyperplasia, and increased heart/body weight ratio. As for the previous mouse model, no indication of hypertrophy was detected. Interestingly, these cardiac defects, together with the consequent ventricular dysfunction, were slightly more pronounced in *Charme*^Δint^ females than in males, for which a similar trend was observed later with age. The upregulation of the fetal Actc1 gene in *Charme*^Δint^ female hearts can anticipate that a sex-specific dysregulated gene program might be correlated to the observed systolic dysfunction. However, a more comprehensive view of *Charme*^Δint^ female and male cardiac transcriptomes will be necessary to assess sex-dependent gene expression contributions to the onset of certain phenotypes when the function of *pCharme* is compromised. If confirmed, the sexual distinction will be of relevance, as the majority of heart failure models have demonstrated more pronounced morbidity associated with the male sex (Du, 2004; Regitz-Zagrosek et al., 2010).

Taken together, our findings provide a deeper insight into the *Charme* mechanism of action in myogenesis and give an intriguing example of how introns may have contributed to the evolution of lineage-specific lncRNAs.

## STAR★Methods

### Key Resources Table

**Table:** 

REAGENT or RESOURCE	SOURCE	IDENTIFIER
**Antibodies**		

MATR3	Bethyl	cat#A300-591A; RRID: AB_495514
PTBP1	Thermo Fisher Scientific	cat#32-4800; RRID: AB_2533082
Mouse IgG	Immunoreagents Inc.	cat#Mu-003-N
Rabbit IgG	Immunoreagents Inc.	cat#Rb-003-N
FUS	Santa Cruz Biotechnology	(4H11) sc-47711; RRID: AB_2105208
Histone H3	Thermo Fisher Scientific	cat#PA5-16183; RRID: AB_10985434
GAPDH	Santa Cruz Biotechnology	(6C5) sc-32233; RRID: AB_627679
Actinin	Santa Cruz Biotechnology	(H-300) sc-15335; RRID: AB_2223809
WGA Alexa Fluor 488-conjugated	Thermo Fisher Scientific	cat#W11261
Donkey anti-rabbit IgG AlexaFluor 647	Invitrogen	cat#A32795; RRID: AB_2762835
Goat anti-rabbit IgG AlexaFluor 488	Invitrogen	cat#A1008
Donkey anti-mouse IgG AlexaFluor 647	Invitrogen	cat#A32787; RRID: AB_2762830
Goat anti-mouse IgG AlexaFluor 405	Invitrogen	cat#A31553; RRID: AB_221604

**Chemicals, Peptides, and Recombinant Proteins**		

Opti-MEM I	Thermo Fisher Scientific	cat#31985047
Protease inhibitor cocktail	Roche	cat#11873580001
DMEM- High glucose	Sigma-Aldrich	cat#D6546
Penicillin/Streptomycin	Sigma-Aldrich	cat#P0781
L-glutamine	Sigma-Aldrich	cat#G7513
PBS	Sigma-Aldrich	N/A
M2 medium	Sigma-Aldrich	cat#M7167
FBS	Sigma-Aldrich	cat#F7524
Transit-X2	Mirus	cat#MIR 6000
Proteinase K	Roche	cat#EO0492
Bradford reagent	Bio-Rad Protein Assay	cat#500-0006
NuPage 4–12% Bis-Tris-Gel	Thermo Fisher Scientific	cat#NP0321BOX
NuPage MES SDS running buffer 20x	Thermo Fisher Scientific	cat#NP0002
NuPage Transfer buffer 20x	Thermo Fisher Scientific	cat#NP0006
4xLaemmli sample buffer	Biorad	cat#1610747
ECL Western Blotting Substrate Pierce	EuroClone	cat#EMP013001
RNase inhibitors	Thermo Fischer Scientific	cat#EO0384
VILO Superscript	Thermo Fisher Scientific	cat#11754050
PrimeScript RT Master Mix	TakaraBio	cat#RR036b
MyTaq DNA Polimerase	Bioline	cat#BIO-21105
PowerUp SYBR-Green MasterMix	Thermo Fisher Scientific	cat#4385612
Dynabeads protein G	Thermo Fischer Scientific	cat#10004D

**Critical Commercial Assays**		

Paris Kit	Thermo Fisher Scientific	cat#AM1921
Neonatal heart dissociation kit	Miltenyi Biotec	cat#130-098-373
Ovation® Ultralow V2 DNA-Seq Library Preparation Kit	NuGEN	cat#0344NB-32
Direct-Zol RNA MiniPrep Kit	Zymo Research	cat#R2050
MAGnify ChIP	Thermo Fisher Scientific	cat#492024
NucleoSpin gel and PCR clean-up kit	TakaraBio	cat#740609.50

**Deposited Data**		

Raw and analyzed ChIP-seq data	This paper	GEO: GSE152308

**Experimental Models: Cell Lines**		

Mouse: cell line C_2_C_12_	ATCC	Strain: C3H

**Experimental Models: Organisms/Strains**		

Mouse: strain C57BL/6	Charles Rivers	Strain code: 027
Mouse: Charme-em1ccpcz (*Charme*^*Δint*^)	This manuscript	N/A

**Oligonucleotides**		

For primers used for RT-qPCR, see Table S1	This paper	N/A
RNA guides used for genome editing, see Table S1	This paper	N/A
siRNAs for cell transfection, see Table S1	This paper	N/A
LNA-Gapmers for cell transfection, see Table S1	This paper	N/A

**Software and Algorithms**		

Guide design tool	Zhang lab	https://zlab.bio/guide-design-resources/T
Proteome Discoverer 1.4 software	Thermo Fisher Scientific	https://www.thermofisher.com/order/catalog/product/OPTON-30810#/OPTON-30810
MODELER 9.18 software	Sali and Blundell, 1993	https://salilab.org/modeller/9.18/release.html
ModeRNA 1.7 software	Rother et al., 2011	http://genesilico.pl/moderna/
Image Lab Software	Biorad	https://www.bio-rad.com/it-it/product/image-lab-software?ID=KRE6P5E8Z
“Jacop” Fiji plugin	NIH	https://imagej.nih.gov/ij/plugins/track/jacop.html
Trimmomatic version 0.322.0.6	Bolger et al., 2014	http://www.usadellab.org/cms/?page=trimmomatic
Bowtie software	Langmead et al., 2009	http://bowtie-bio.sourceforge.net/index.shtml
Samtools rmdup	Li et al., 2009	http://www.htslib.org/
HOMER program	Heinz et al., 2010	http://homer.ucsd.edu/homer/
Bedtools intersect	Quinlan and Hall, 2010	https://bedtools.readthedocs.io/en/latest/
THOR software	Allhoff et al., 2016	https://www.regulatory-genomics.org/thor-2/basic-intrstruction/
Cluster 3.0 software	de Hoon et al., 2004	http://bonsai.hgc.jp/∼mdehoon/software/cluster/software.htm
Java Treeview software	Saldanha, 2004	http://jtreeview.sourceforge.net/
Vevo 2100 Imaging System	VisualSonics, Inc.	https://www.visualsonics.com/product/imaging-systems/vevo-2100
ImageJ software	NIH	https://imagej.nih.gov/ij/download.html
STRING	Szklarczyk et al., 2019	https://string-db.org

### Resource Availability

#### Lead contact

Further information and requests for resources and reagents should be directed to and will be fulfilled by the Lead Contact, Monica Ballarino (monica.ballarino@uniroma1.it)

#### Materials availability

The *Charme*^Δint^ mouse line generated in this study has been deposited to the EMMA repository.

#### Data and code availability

Data that support the findings of this study have been deposited in NCBI Gene Expression Omnibus (GEO) database (https://www.ncbi.nlm.nih.gov/geo/query/acc.cgi?acc=GSE152308). UCSC genome browser session displaying ChIP-seq tracks: https://genome.ucsc.edu/cgi-bin/hgTracks?db=mm10&lastVirtModeType=default&lastVirtModeExtraState=&virtModeType=default&virtMode=0&nonVirtPosition=&position=chr12%3A56694976%2D56714605&hgsid=967667035_S4FWMmR36Y5qu4cBwgU2V47v0i0v.

### Experimental Model and Subject Details

#### Mouse models and care

Mice with specific deletion intron-1 were generated in a C57BL/6N background using a CRISPR genome-editing system (Yang et al., 2015). For this purpose, *in vitro* transcribed Cas9 mRNA and sgRNAs (designed with https://zlab.bio/guide-design-resources) respectively at the 5′ and the 3′ of intron-1 (Table S1), were injected into the cytoplasm of fertilized eggs of the C57BL/6N mice in M2 medium (Sigma-Aldrich, MO, USA). The correct genome editing was confirmed by PCR amplification in the founder mouse with the oligo listed in Table S1. The mutant allele was backcrossed for four generation to obtain homozygous animals. Animals were bred and maintained in respect to housing, nutrition, and care according to the animal welfare rules of the Czech Republic. 2-6 weeks old male and female mice were used for gene expression, subcellular fractionation and DNA-DNA FISH experiments while 6-10-12-16-36-58 weeks old male and female mice were used for morphometric and histological analyses. All experiments were approved by the Institutional Animal Use and Care Committee (approval no. 115-2016) and were carried out in accordance with the law.

#### Cell culture

C_2_C_12_ murine myoblasts were cultured in a humidified incubator at 37°C and 5% CO_2_ in growth [DMEM high glucose (Sigma-Aldrich, Saint Louis, MO, USA), 20% FBS (Sigma-Aldrich)] or differentiation media [DMEM high glucose (Sigma-Aldrich, Saint Louis, MO, USA), 0.5% FBS (Sigma-Aldrich)] with the addition of 1x L-glutamine (Sigma-Aldrich) and 2x penicillin-streptomycin (Sigma-Aldrich). See Key Resources Table for details.

### Method Details

#### Cell transfection

Cells (150x10^3^) were plated in 35 mm plates and transfected 24 hr later with 75 nM of LNA GapmeRs (Exiqon) or si-SCR/si-MATR3 (50 nM) or si-SCR/si-PTBP1 (100 nM) siRNAs (SMARTpool, Dharmacon) in 3 μl/ml of Transit-X2 transfectant (Mirus) and 100 μl/ml of Opti-MEM (Thermo Fisher Scientific), according to manufacturer’s specifications. Details on the GapmeRs and siRNAs used are reported in Table S1. See Key Resources Table for reagents details.

#### RNA Affinity Purification-Mass Spectrometry (RAP-MS)

Forty-five 5′-biotinylated (90-mer long) DNA oligonucleotides antisense to intron-1 were designed and synthetized. 200 million differentiated C_2_C_12_ cells were harvested in PBS and used for RAP according to McHugh et al. (2015), with minor modifications. Briefly, cells were UV-crosslinked at 254 nm on ice using a Spectrolinker UV Crosslinker and lysed in 1 mL Lysis Buffer 1 [10 mM HEPES pH7.2, 20 mM KCl, 1.5 mM MgCl_2_, 0.5 mM EDTA, 1 mM Tris (2-carboxyethyl) phosphine (TCEP), 0.5 mM PMSF]. After centrifugation (3,300 × g for 10 min), pellets were resuspended in 1 mL Lysis Buffer 1 with 0.1% dodecyl maltoside (DDM) and dounced 20 times using a glass homogenizer with the small clearance pestle (Kontes). Released nuclei were pelleted by centrifugation (3,300 × g) and resuspended in 550 μl Lysis Buffer 2 [20 mM Tris pH 7.5, 50 mM KCl, 1.5 mM MgCl_2_, 2 mM TCEP, 0.5 mM PMSF, 0.4% sodium deoxycholate, 1% DDM, and 0.1% N-lauroylsarcosine]. Lysate was sonicated, treated with 2 M Urea and 1.25 mM DTT and precleared. Extract was then incubated at 67°C for 2 h with biotinylated antisense probes (10 μg), specific for *pCharme* or U1 RNAs, before adding streptavidin-coated beads (Promega). After extensive bead washing, RNA was eluted through NLS elution buffer [20 mM Tris HCl pH 8, 10mM EDTA, 2% NLS, 2.5mM DTT] for enrichment analysis by qRT-PCR, whereas proteins were eluted using Benzonase Elution buffer [20mM Tris HCl pH 8, 0.05% NLS, 2 mM MgCl_2_, 0.5 mM DTT] for MS analysis.

#### Mass spectrometry (MS) analysis

*pCharme* and U1 snRNA co-purified proteins were precipitated by adding TCA to a final 10% concentration to protein elution sample and incubated at 4°C overnight. The day after, the samples were centrifugated (16,000 x g for 30 min) and the protein pellets were washed with 1 mL of cold acetone. Pellets were dried in open tube on bench and the lyophilized proteins stored at −20°C. Samples were digested in LysC (Wako SAG4751)/Trypsin (Promega) solution and C18 desalted. MS analysis was performed in the LTQ Velos Pro/Nanocolumn Acclaim (PepMap 25cm) mass spectrometer and peptide mixtures were separated with 2 h gradient long (Top20CID). For data analysis, proteins were identified by database searching using SequestHT/Percolator (Thermo Fisher Scientific) with Proteome Discoverer 1.4 software (Thermo Fisher Scientific) against the Reference Proteome Mouse_2016_07,49153 entries. Peptides were filtered with a false discovery rate (FDR) at 1% and 2 unique peptides minimum/proteins. Selection of the final protein candidates was performed by applying the following criteria. The original protein list (Table S2, sheet “raw”) was searched for cytoplasmic and keratin contaminants, which were manually removed. Proteins displaying a score different than zero in all the three MS replicates were further selected. Ranking was then generated on the protein average scores that was unbiasedly computed by the MS facility on the number and the coverage of the retrieved peptides. *pCharme* interactors displaying at least a 3-fold enrichment score over U1 were included in the final list (Table S2, sheet “filtered”).

#### Crosslinking Immunoprecipitation (CLIP) assay

Cells were UV-crosslinked at 4,000 μJ. using a Spectrolinker UV Crosslinker and the nuclear extracts collected according to Rinn et al. (2007), with minor modifications. Nuclear pellet was resuspended in 1 mL of NP40 lysis buffer [50 mM HEPES pH 7.5, 150 mM KCl, 2 mM EDTA, 1 mM NaF, 0.5% (v/v) NP40, 0.5 mM DTT, complete EDTA-free protease inhibitor cocktail (Roche)] and nuclear membrane were lysed with dounce homogenizer (20 strokes). The nuclear lysate diluted to a final concentration of 1 mg/ml. 30 μl of Dynabeads Protein G magnetic particles (Invitrogen) per ml of nuclear lysate were washed twice with 1 mL of PBS-Tween (0.02%), resuspended with 10 μg of MATR3 (Bethyl) or IgG a specific antibodies (Immunoreagents Inc.) and incubated for 1 h at room temperature. Beads were then washed twice with 1 mL of PBS-T and incubated with nuclear extract overnight at 4°C. Beads were washed three times with 1 mL of HighSalt NP40 wash buffer [50 mM HEPES-KOH, pH 7.5, 500 mM KCl, 0.05% (v/v) NP40, 0.5 mM DTT, complete EDTA-free protease inhibitor cocktail (Roche)], twice with 1 mL of polynucleotide kinase (PNK) Buffer [50 mM Tris-HCl pH 7.5, 50 mM NaCl, 10 mM MgCl_2_, 5 mM DTT] and resuspended in 100 μl of NP40 lysis buffer. 75 μl were collected for RNA analysis: an equal volume of 2x Proteinase K Buffer (100 mM Tris-HCl, pH 7.5, 150 mM NaCl, 12.5 mM EDTA, 2% (w/v) SDS) was added, followed by the addition of Proteinase K (Roche) to a final concentration of 1.2 mg/ml and incubated for 30 min at 55°C. The RNA was recovered and analyzed through qRT-PCR. 25 μl were heated at 95°C for 5 min and the supernatant collected and resuspended in Protein elution buffer [4x Laemmli sample buffer (BioRad)] with DTT 50 mM and analyzed by Western Blot.

See Key Resources Table for details.

#### Modeling of MATR3/PTBP1/pCharme interactions

The hypothetical binding sites on the *pCharme* sequence were identified by scanning for the MATR3 consensus sequence [CT][CT]TTTCT.TTT as reported in Uemura et al. (2017). The PTBP1 binding sites on *pCharme* intron-1 were obtained by retrieving PAR-CLIP seq data from the POSTAR database (Hu et al., 2017) and using the consensus sequence T[CT]T[CT][CT] reported in Xue et al. (2009). The RRM1 and RRM2 domains of mouse PTBP1 were obtained through comparative modeling with the MODELER 9.18 software (Sali and Blundell, 1993) using the PDB structures 2AD9 and 2ADB, respectively, as templates while the RRM3 and RRM4 domains were built using the PDB structure 2ADC as a template. Loops joining PTBP1’s globular domains were not modeled. A model of mouse MATR3 RRM1 and RRM2 domains bound to RNA was obtained through comparative modeling with MODELER using the 2ADC structure as a template. The interaction between MATR3 PRI (PTBP1 RRM interaction motif) and PTBP1 RRM2 was modeled using the structural information from the PDB structure 3ZZY. The intronic portions were modeled using the ModeRNA 1.7 software (Rother et al., 2011).

#### Protein analyses

Total protein extracts were prepared by resuspending the cell pellets in 50-100 μL of Protein Extraction Buffer [100 mM Tris pH 7.5, 1 mM EDTA, 2% SDS, 1x PIC]. The mix was incubated 20 min on ice and centrifuged at 15,000 x g for 15 min at 4°C. Nucleoplasm/Chromatin/Cytoplasm fractionation was performed as follows. C_2_C_12_ cells were lysed in cytoplasmic lysis buffer [10 mM HEPES pH 7.9, 0.34 M sucrose, 3 mM CaCl_2_, 2 mM MgAc, 0.1 mM EDTA, 1 mM DTT, 0.5% (v/v) NP-40, 100 x Protease inhibitor cocktail (PIC)]. The lysate was centrifuged 15 min at 2,600 x g and the cytoplasmic fraction was collected. Intact nuclei were washed with cytoplasmic buffer without NP-40 and pelleted. Nuclei were then lysed with nuclear buffer [20 mM HEPES pH 7.9, 3 mM EDTA, 10% glycerol, 150 mM KAc, 1.5 mM MgCl_2_, 1 mM DTT, 0.1% NP-40, 100x PIC] and sonicated on ice 2 cycles at low intensity (10 repeats, 30 s ON-30 s OFF) using a Bioruptor sonicator. The nucleoplasmic fraction was then cleared by centrifugation 30 min at 27,000 x g. The chromatin pellet was resuspended in nuclease incubation buffer [150 mM HEPES pH 7.9, 1.5 mM MgCl_2_, 150 mM KAc, 10% glycerol, 100x PIC], sonicated on ice 10 cycles at high intensity (10 repeats, 30 s ON-30 s OFF).

Protein concentration was measured by spectrophotometric quantification using the Bradford reagent (Bio-Rad Protein Assay), following manufacturer’s instructions. For Western Blot analysis, proteins (15-30 μg) were loaded on 4%–12% bis-tris-acrylamide gel (Thermo Fisher scientific) and transferred to a nitrocellulose membrane. The membrane was blocked in 5% milk and hybridized with the specific antibodies overnight at 4°C at the appropriate dilutions, according to manufacturers’ instructions (see Table S1 for details). After three washes in TBST, the filter was hybridized with the corresponding secondary antibody for 1 h at room temperature. Protein detection was carried out with Long Lasting Chemiolominescent Substrate (EuroClone) using ChemiDoc MP System and images were analyzed using Image Lab Software (BioRad).

See Key Resources Table for details.

#### RNA analyses

Total RNA was isolated from cultured cells and mice tissues. For tissues, hearts from WT and mutant mice were mechanistically reduced to a pulp and resuspended in 500 μL of TRI Reagent (Zymo Research). Samples were centrifuged for 30 min at 16,000 x g at 4°C to collect the supernatant. RNA was isolated using Direct-zol RNA MiniPrep Kit (Zymo Research) according to manufacturer’s instructions and quantified by Nanodrop (Thermo Scientific). From C_2_C_12_ cells, total RNA was isolated using TRI Reagent (Zymo Research) followed by column purification using Direct-zol RNA MiniPrep Kit (Zymo Research) and quantified by Nanodrop (Thermo Scientific). For semiquantitative and quantitative RT-PCR analyses, RNA (0.5-1.0 μg) was reverse transcribed using PrimeScript Reagent Kit (Takara), according to manufacturer’s instructions. Amplification by PCR was carried out using Mytaq (Bioline) (RT-PCR) or PowerUp SYBR-Green MasterMix (Thermo Fisher Scientific) (RT-qPCR) reagents. See Table S1 for oligo and Key Resources Table for reagents details.

#### Nucleoplasm/Chromatin/Cytoplasm fractionation

C_2_C_12_ cells were lysed in 1X RNA lysis buffer [2x RNA lysis buffer: 0.28 M NaCl, 3 mM MgCl_2_, 20 mM TrisHCl pH 7.5, 1% NP40, 100 x PIC, 200 x RNase inhibitor]. The lysate was incubated 10 min on ice and 1 volume of RNA lysis buffer/sucrose [2 x RNA lysis buffer, 24% (w/v) sucrose] was added. After 10 min of centrifugation at 15,000 x g at 4°C the supernatant was collected as cytoplasmic fraction. All the sucrose was removed with a syringe and the nuclear pellet was washed with 1 x RNA lysis buffer and 1 volume of RNA lysis buffer/sucrose. After 10 min of centrifugation (15,000 x g at 4°C) the supernatant was removed with a syringe and the nuclear pellet resuspended in Buffer 1 [75 mM NaCl, 20 mM TrisHCl pH 7.9, 0.5 mM EDTA, 0.85 mM DTT, 0.1 mg/ml yeast tRNA, 50% (v/v) Glycerol, 100 x PIC, 200 x RNase inhibitor] and 10 volumes of Buffer 2 [0.3 M NaCl, 20 mM HEPES pH 7.6, 0.2 mM EDTA, 1 mM DTT, 0.1 mg/ml yeast tRNA, 7.5 mM MgCl_2_, 1M UREA, 1% NP40, 200X RNase inhibitor) was added. The lysate was vortexed and incubated 10 min on ice. The nucleoplasmic fraction was then cleared by centrifugation (10 min at 15.000 x g) at 4°C. The chromatin pellet and the cytoplasmic/nucleoplasmic fractions were directly resuspended in TRI Reagent (Zymo Research) for RNA extraction.

Nucleus/Cytoplasm fractionation on murine hearts was performed using neonatal heart dissociation kit (Miltenyi Biotec), according to the manufacturer’s instructions. At the end of the dissociation program, 7.5 mL of DPBS with 20% FBS was added to the cell suspension and the mixed solution was applied to a MACS SmartStrainer (70 μm) and placed on a tube. After 5 min of centrifugation at 600 x g, cellular pellet was washed with 10 mL of DPBS and the solution was centrifuged 5 min at 600 x g. The supernatant was completely removed, and the cellular pellet was treated with PARIS™ kit (Thermo Fisher Scientific), according to manufacturer’s instructions.

See Key Resources Table for details.

#### Immunofluorescence, RNA and DNA-FISH

Imaging experiments were carried out according to Santini et al. (2021), with minor modifications. Briefly, MATR3/PTBP1/*pCharme* co-staining was conducted by performing immunofluorescence (IF) for MATR3 and PTBP1 before *pCharme* Fluorescent *in situ* hybridization (FISH) using fluorescent (Fluorescein or Cy3)-conjugated synthetic DNA oligonucleotides (RNA-FISH) (see Table S1 and Ballarino et al., 2018). For RNA FISH, fixed cells were permeabilized in 0.5% Triton (10 min at 4°C) and incubated O.N. at 37°C with fluorescent DNA probes diluted in hybridization buffer [10% dextran sulfate, 2 × SSC, 10% formamide, 2 mM ribonucleoside-vanadyl complex]. For immunofluorescence, primary antibodies against PTBP1 and MATR3 and specific secondary antibodies were used at the appropriate dilutions (see Key Resources Table). Potential multichannel crosstalk between MATR3 and *Charme* signals was avoided by using two different secondary antibodies conjugated with Alexafluor 488 (Figures 2A and 3D) or Alexafluor 647 (Figures 3G and S3A). For DNA-FISH on histological muscle cryosections, after permeabilization with triton buffer [0.5% Triton 100X/PBS], a mild pepsin digestion [Pepsin 0.1%/0.1M HCl] was applied before denaturation in order to reduce the autofluorescence deriving from protein components. *Charme* and *nctc* genomic regions were visualized by nick translated BAC clones labeled with dUTP-cyanine3 (RP23-46J16, *Charme* locus) and dUTP- 5-Fluorescein conjugated (RP23-352B6, *nctc* locus) (Enzo Life-Sciences). Images were acquired as Z stacks (200 nm path) by confocal microscopy equipped with a Confocal Imager (CREST X-LIGHT) spinning disk, a 60X NA 1.35 oil (UPLANSApo) and a CoolSNAP Myo CCD camera (Photometrics), which allow to obtain an optimal optical resolution (1 pixel = 75 nm). For post-acquisition studies, FIJI software was used. In particular, interallelic distance (3D-distance) was taken on Z stacks images by Spot Distance plugin and then normalized respect to nuclei diameter (Normalized 3D Distances/d, d = major axis+minor axis/2). Nucleoplasmic MATR3 fluorescence intensity in GAP-SCR or GAP-1 condition was quantified as mean intensity (total signal intensity normalized for the nuclei area) by using a Regions Of Interest (ROI) mask that exclude the *pCharme*/MATR3 colocalized areas for fluorescence measurement. Quantification of colocalized *pCharme*/MATR3 and MATR3 /*pCharme* signals was performed on the nuclear areas and measured as percentage ratio (%) of fluorescence intensity of colocalized signals respect to total nuclear signals [(raw integrated density of colocalized areas / raw integrated density of total nuclear signal) ^∗^100]. The Pearson’correlation coefficient was calculated by performing 3D analysis on Z stacks using the “Jacop” Fiji plugin.

#### Chromatin immunoprecipitation (ChIP)-seq

ChIP experiments were performed on chromatin extracts according to manufacturer’s protocol (MAGnify ChIP, Thermo Fisher Scientific). Sheared chromatin from GAP-SCR and GAP-1 treated myotubes, was incubated O.N. with 5 μg of polyclonal anti-MATR3 (Bethyl) or rabbit IgG antibodies. Immunoprecipitated DNA samples were quantified by Qubit 2.0 Fluorometer (Invitrogen, Carlsbad, CA). Ovation® Ultralow V2 DNA-Seq Library Preparation Kit (NuGEN, Redwood City, CA) was used for library preparation following the manufacturer’s instructions. Final libraries were checked with both, Qubit 2.0 Fluorometer (Invitrogen, Carlsbad, CA) and Agilent Bioanalyzer DNA assay or Caliper (PerkinElmer, Waltham, MA).

For both GAP-SCR and GAP-1 experiments, IgG, Input and two MATR3 IP libraries were prepared and sequenced on HiSeqv4 (Illumina, San Diego, CA) at the Institute of Applied Genomics (IGA; Udine, Italy), yielding an average of about 35 million 50 nucleotides long single-end reads per sample. Quality trimming was performed using Trimmomatic software with parameters LEADING:3 TRAILING:3 SLIDINGWINDOW:4:15 MINLEN:40 (Bolger et al., 2014). Reads were aligned to mouse mm10 genome using Bowtie software (Langmead et al., 2009) with parameters–best–strata -m 1. PCR duplicates were removed using samtools rmdup (Li et al., 2009). Manual inspection of reads alignment revealed that reads from IP samples tended to have a broad enrichment regime, i.e., they did not form sharp peaks. For both GAP-SCR and GAP-1 experiments we used HOMER getDifferentialPeaksReplicates.pl program (Heinz et al., 2010) to call MATR3-enriched regions by comparing IP samples versus their corresponding Input samples (q-value < 0.1); to allow enriched regions with variable length we launched the program twice, once with -style histone parameter and the other with -region -size 260 parameter. MATR3-enriched regions obtained with both parameters were identified using Bedtools intersect (Quinlan and Hall, 2010); this list of overlapping regions was supplemented with regions obtained using only one of the two parameters. IgG-enriched regions were called using HOMER findPeaks by comparing IgG samples with their corresponding Input samples with both -style histone and -region -size 260 parameters. IgG-enriched regions from both GAP-SCR and GAP-1 experiments were pooled together and used to filter off MATR3-enriched regions close to them using Bedtools window with parameters -v -w 500. This way we obtained two lists of MATR3-enriched regions, one for the GAP-SCR experiment and the other for the GAP-1 experiment. Genome Ontology term enrichment analysis on these two lists was performed using HOMER annotatePeaks.pl. Regions differentially enriched between GAP-SCR and GAP-1 experiments were called using THOR software (Allhoff et al., 2016), setting the IgG-enriched regions as dead zones. Bedtools intersect was used to retain only those differentially enriched regions which overlapped enriched regions called by HOMER software. Read density profiles for 1000 bp long regions around differentially enriched regions center were computed using HOMER annotatePeaks.pl with parameters -size 1000 -hist 25 -ghist after pooling alignment files from IP replicates. Read density profiles were clustered with Cluster 3.0 software (de Hoon et al., 2004), using centered Pearson correlation to calculate distances via complete linkage method. The resulting clustered heatmap was visualized using Java Treeview software (Saldanha, 2004). For ChIP-seq validation, the candidate amplicons were amplified by qPCR using the SYBR Green reagent (Applied Biosystem). Details on the oligonucleotides used for the different amplifications are reported in Table S1. A standard curve was generated for each primer pair testing 5-point dilutions of input sample and used for the absolute quantification. The IgG background was then subtracted, and data expressed as percentage of input chromatin (Input%). See Key Resources Table for details.

#### Echocardiography

The echocardiographer was blinded to the phenotypes. Transthoracic ultrasound imaging was acquired using the Vevo 2100 Imaging System (VisualSonics, Inc.) with a 30 MHz transducer (MS400) operating at a frequency that provides highly reliable and reproducible image quality. Echocardiography was performed on anaesthetized mice and during imaging, the concentration of anesthesia (1%–2% isoflurane) was controlled to maintain a heart rate of 450-500 beats/min. Left ventricular function was assessed by M-mode scanning of the left ventricle chamber, standardized by two-dimensional, short-axis views of the left ventricle at the mid papillary muscle level. Wall thickness and internal dimensions of the left ventricle at diastole and systole (LVID;d and LVID;s, respectively) were measured in at least three beats from each projection and averaged. The fractional shortening (FS) of the left ventricle was calculated as FS% = [(LVID;d-LVID;s)/LVID;d]x100, representing the relative change of the left ventricular diameters during the cardiac cycle. The mean FS of the left ventricle was determined by the average of FS measurements of the left ventricular contraction over three beats. p values were calculated by two-way ANOVA.

#### Morphometric and histological assessment

At the end of the study, standard morphometric measurements were obtained including body and heart weights as well as tibia length. In some cases, isolated hearts were quickly perfused with ice-cold cardioplegic solution [30 mM KCl in PBS] to arrest the heart in diastole. Such hearts were fixed with 4% formaldehyde, paraffin-embedded and serially sectioned (3 μm slices). For morphometric analysis of cardiomyocytes, paraffin sections were deparaffinized and incubated with wheat germ agglutinin (WGA; Alexa Fluor 488-conjugated; Life Technologies) for 30 min at 37°C in dark, briefly washed with PBS, stained with DAPI and then mounted with coverslips using DAKO fluorescent mounting medium (Dako). The images were captured with a Zeiss model microscope (Axio Imager.z2) and analyzed with NIH ImageJ software. p values were calculated by Student`s t test.

### Quantification and Statistical Analysis

No statistical analysis was used to predetermine sample size and the suitability of statistical approaches. Quantifications were performed from at least three independent experiments and quantified blindly. Sample sizes, statistical tests and p values are indicated in figure legends and Method Details. All the quantitative data are presented as mean ± SD or ± SEM.

## Supplemental Information

Document S1. Figures S1–S4

Table S1. List and Sequences of the Oligonucleotides, GapMers, siRNAs, Antibodies and DNA Probes Used, Related to Figures 1, 2, 3, and 4

Table S2. Mass Spectrometry Results from *pCharme* and U1 snRNA RNA Antisense Purification (RAP), Related to Figure 1 The “Filtered” sheet shows the list of the filtered protein interactors in *pCharme* and U1 snRNA samples. The average score, together with Score, Coverage, Peptides sum, and peptide-spectrum match (PSM) values for each mass spectrometry replicate are reported.

Table S3. U1 snRNA and *pCharme* Protein Interactors, Related to Figure 1

Document S2. Article plus Supplemental Information
