# Supplementary material for: Intronic Determinants Coordinate Charme lncRNA Nuclear Activity through the Interaction with MATR3 and PTBP1
Source: Cell Rep. 2020 Dec 22;33(12):108548. doi: 10.1016/j.celrep.2020.108548 (PMC7773549; doi:10.1016/j.celrep.2020.108548)
Supplement: Document S1. Figures S1–S4 [file mmc1.pdf]

Cell Reports, Volume *33*

Supplemental Information

Intronic Determinants Coordinate *Charme* lncRNA Nuclear Activity through the Interaction with MATR3 and PTBP1

Fabio Desideri, Andrea Cipriano, Silvia Petrezselyova, Giulia Buonaiuto, Tiziana Santini, Petr Kasparek, Jan Prochazka, Giacomo Janson, Alessandro Paiardini, Alessandro Calicchio, Alessio Colantoni, Radislav Sedlacek, Irene Bozzoni, and Monica Ballarino

Figure S1

A

5'-3' SPLICE SITES

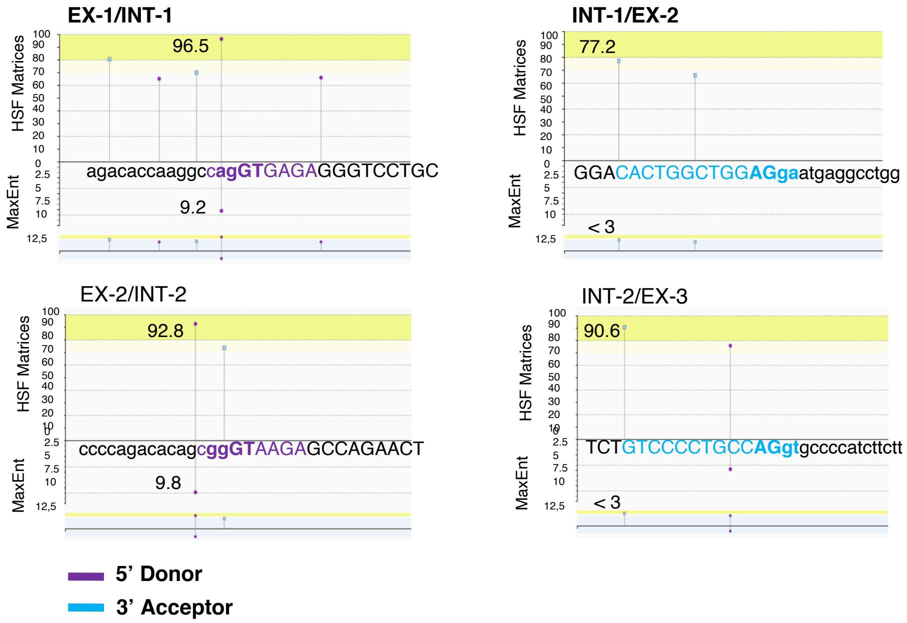

B

BRANCH POINTS

INTRON-1

GCATGGCTAAGACTGAGTCCAGGTGT
GGCTCAGGCTTTGTGACACTCTTTTTT
TTTTTTTTTTTTTTCTGTTTGCCCT
GGACACTGGCTGGAGgaatgaggcctgg

Branch Point Motif (CV= 91.07)

INTRON-2

ATGGGCTCTGGGACTCTTGTGAGA
TTGATGAAGGCTGATGAGGCTGC
TCTGTCCCCTGCCAGgtgccccatcttctt

Branch Point Motif (CV= 84.96)

C

Molecular Function

Term ID	Description	Background genes	FDR	Matching proteins
GO:0003723	**RNA binding**	986	0.00044	Matr3,Pcbp2,Pcbp4,Ptbp1,Ptbp2
GO:0003729	mRNA binding	202	0.0010	Pcbp4,Ptbp1,Ptbp2
GO:0036002	pre-mRNA binding	33	0.0011	Ptbp1,Ptbp2
GO:0003697	single-stranded DNA binding	97	0.0067	Pcbp2,Ptbp1

Biological Process

Term ID	Description	Backgroun d genes	FDR	Matching proteins
GO:0033119	**negative regulation of RNA splicing**	31	6.49e-05	Pcbp4,Ptbp1,Ptbp2
GO:0075522	IRES-dependent viral translational initiation	10	0.0013	Pcbp2,Ptbp1
GO:0048025	negative regulation of mRNA splicing, via spliceosome	25	0.0023	Pcbp4,Ptbp1
GO:0008380	RNA splicing	310	0.0065	Prpf38a,Ptbp1,Ptbp2
GO:0006397	mRNA processing	384	0.0109	Prpf38a,Ptbp1,Ptbp2
GO:0010608	posttranscriptional regulation of gene expression	392	0.0109	Matr3,Pcbp4,Ptbp1
GO:0043488	regulation of mRNA stability	80	0.0111	Pcbp4,Ptbp1

D

MATR3-CLIP

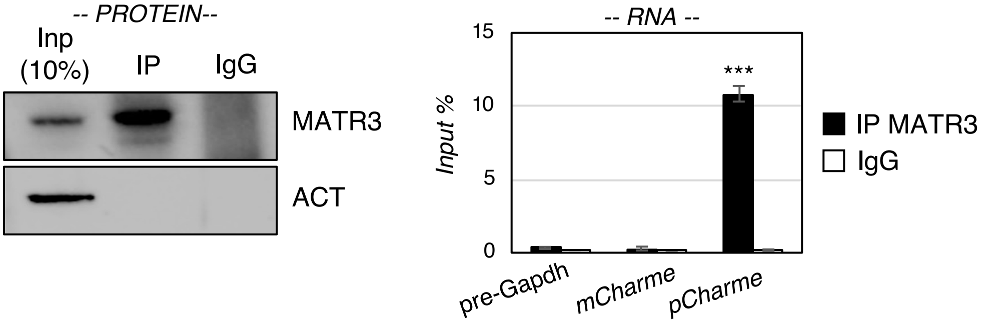

Figure S1. *pCharme* intronic features and protein interactors, related to Figure 1: (**A, B**) *In silico* analyses performed with the Human Splicing Finder (HSF) 3.1 tool (Desmet et al., 2009) to identify splicing motifs within *Charme* primary transcript. Graphical representation of predicted donor (violet)/acceptor (light blue) splice sites (**A**) and branch point motifs (red) (**B**) are shown together with their consensus values (CV). CVs span from 0 to 100 for HSF (threshold = 65), -20 to +20 for MaxEnt (threshold = 3) and from 0 to 100 for branch point motifs. Every signal with a score above the threshold is considered as donor (violet) or acceptor (light blue) splice site. HSF scores above 80 are associated to strong splice sites. Intron-exon junctions and the adenines at the branch point are highlighted in bold. (**C**) Gene ontology (GO) term enrichment analysis performed on the top eight *pCharme* protein interactors using STRING (Szklarczyk et al., 2019). The topmost Molecular Function (top) and Biological Process (bottom) GO categories are shown. (**D**) MATR3 cross-linked RNA immunoprecipitation (CLIP) assay performed on nuclear extracts from differentiated myotubes. Left: representative image of western blot analysis performed to test MATR3 recovery in the IP and IgG samples. Actinin (ACT) protein serves as negative control. Right: qRT-PCR quantification of *pCharme* and *mCharme* transcripts recovery in the IP and IgG samples. GAPDH precursor (pre-GAPDH) RNA serves as negative control. Values are expressed as input percentage (%) and represent the mean±SD of three biological triplicates. See **Table S1** for primer sequences and **Key Resources Table** for antibodies.

Data information ***P < 0.001, unpaired Student's t-test.

Figure S2

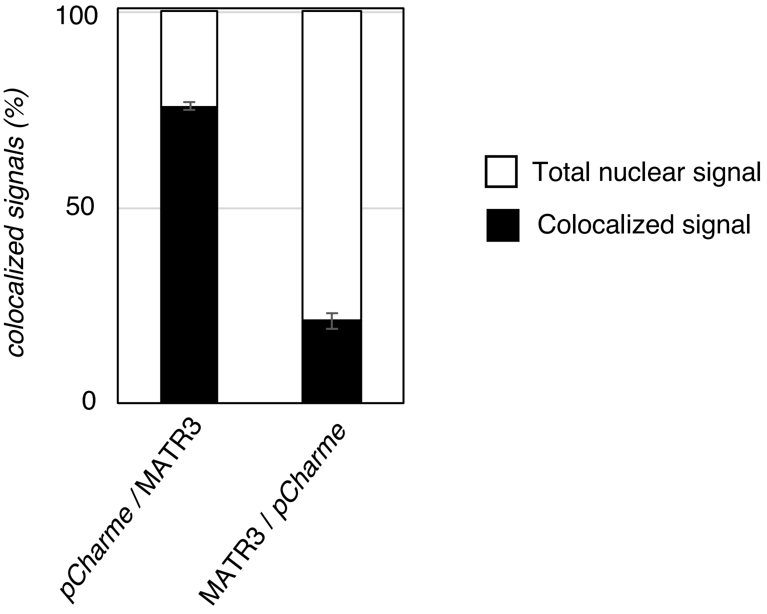

Figure S2. *pCharme* and MATR3 spatial colocalization, related to Figure 2: Histogram representing the ratio (%) of *pCharme*/MATR3 and MATR3/*pCharme* colocalized signals (black) in respect of total nuclear signals (white). Quantitative analysis was performed on a total of 61 nuclei from 3 independent experiments.

Figure S3

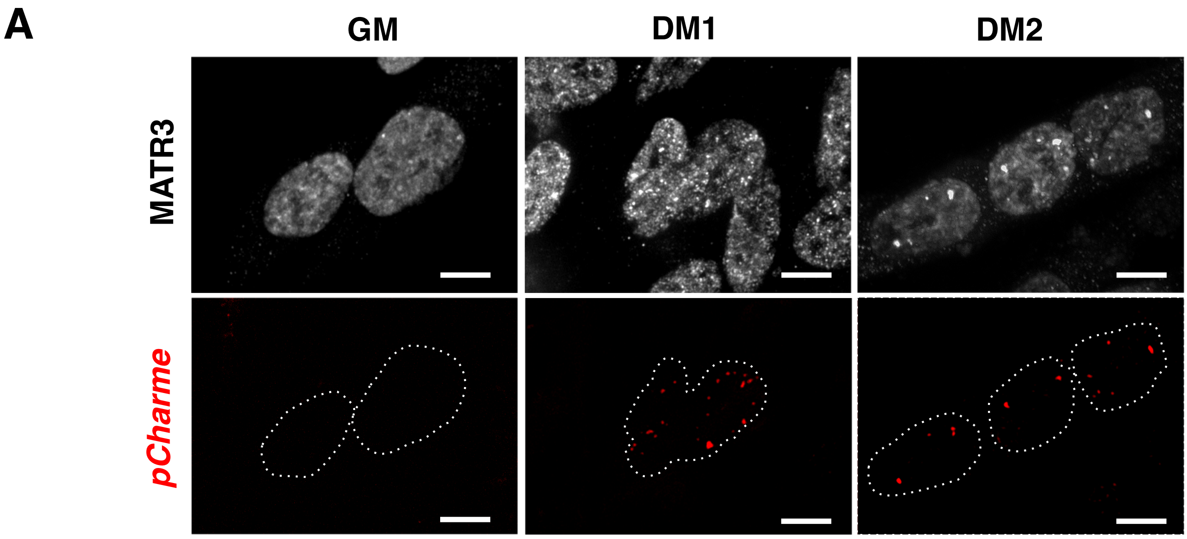

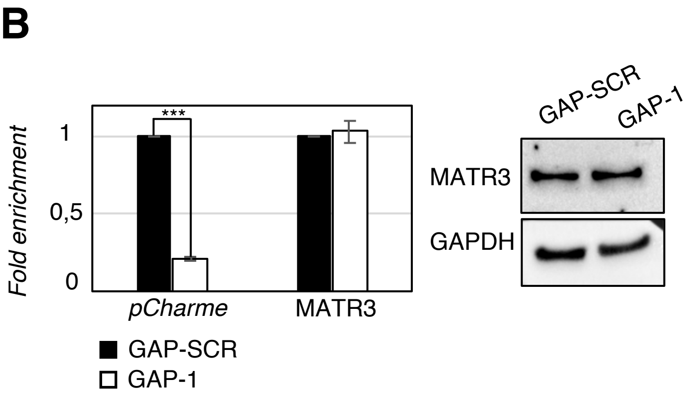

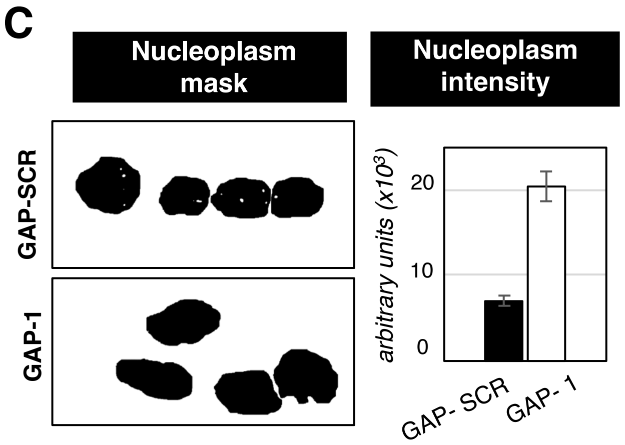

D

GAP-SCR ontology term enrichment

Annotation	Overlap (regions)	Log(Ratio Enrichment)	Log(P-value)
**CpGIsland**	2365	2.67	-3289.69
**exons**	5797	1.26	-1708.94
**protein-coding**	5625	1.3	-1707.34
**promoters**	2335	1.54	-1322.55
**LTR**	3712	-0.89	982.6
**LINE**	2683	-1.69	4102.37
**LINEIL1**	2068	-1.79	4212.22
**intergenic**	9359	-0.83	13379.91

GAP-1 ontology term enrichment

Annotation	Overlap (regions)	Log(Ratio Enrichment)	Log(P-value)
**CpGIsland**	136	2.69	-145.64
**exons**	330	1.28	-79.76
**protein-coding**	320	1.29	-74.21
**promoters**	134	1.58	-64.61
**LTR**	175	-1.11	61.29
**LINE**	130	-1.77	189.88
**LINEIL1**	94	-1.89	194.71
**intergenic**	365	-0.93	668.8

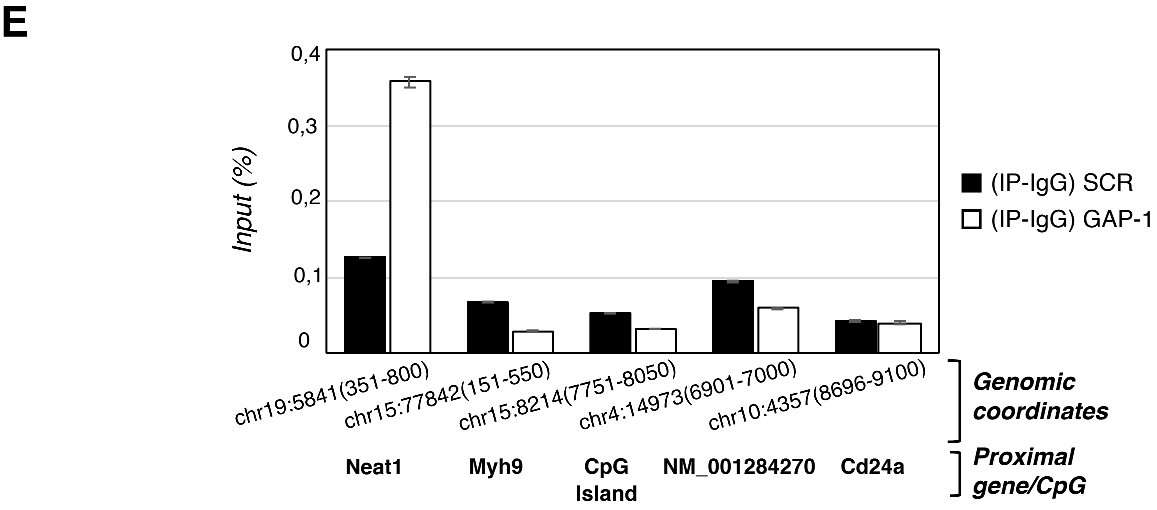

Figure S3. ***In vitro*** **analysis of MATR3/*pCharme* interaction, related to Figure 3:** (**A**) Representative images of MATR3 immunofluorescence (grey) and *pCharme* RNA-FISH (red) performed in differentiating C_2C_{12} cells at the indicated time points. Dashed lines indicate the edge of the nuclei. GM= growth medium, DM=differentiation medium. Scale bar: 10 µm (**B**) Left: qRT-PCR quantification of *pCharme* and MATR3 RNA levels in 2-days differentiated myotubes treated with GAP-SCR or GAP-1. Data were normalized to GAPDH mRNA and represent mean±SEM of three independent experiments. Right: Western blot analysis of MATR3 protein in 2-days differentiated myotubes treated with GAP-SCR or GAP-1. GAPDH protein was used as a calibrator. See **Table S1** for primer sequences and **Key Resources Table** for antibodies. (**C**) Left: Regions Of Interest (ROI) mask selected to quantify MATR3 fluorescence intensity in the nucleoplasm of myotubes as shown in **Figure 3G**. In GAP-SCR sample the *pCharme*/MATR3 colocalized areas were excluded for the fluorescence intensity measurement. Right: scatter dot blot representing mean intensity ± SD (total signal intensity normalized for the nuclear area) of MATR3 signals in the nucleoplasm of GAP-SCR and GAP-1 treated myotubes (shown in **Figure 3G**). (**D**) Genome Ontology term enrichment analyses of the regions bound by MATR3 in GAP-SCR (left) *vs* GAP-1 (right) conditions. Enriched regions were called independently for both the experiments. (**E**) MATR3 ChIP-seq validation of the differentially bound regions by qPCR analysis performed on a biological independent replicate. Data were normalized and represented as percentage (%) of Input in GAP-SCR *vs* GAP-1 treated myotubes. Region with increased binding: Neat1; regions with decreased binding: Myh9, CpG Island and NM_001284270. Not differentially bound region: Cd24a. See **Table S1** for oligo sequences.

Data information: $***P < 0.001$, unpaired Student's t-test.

Figure S4

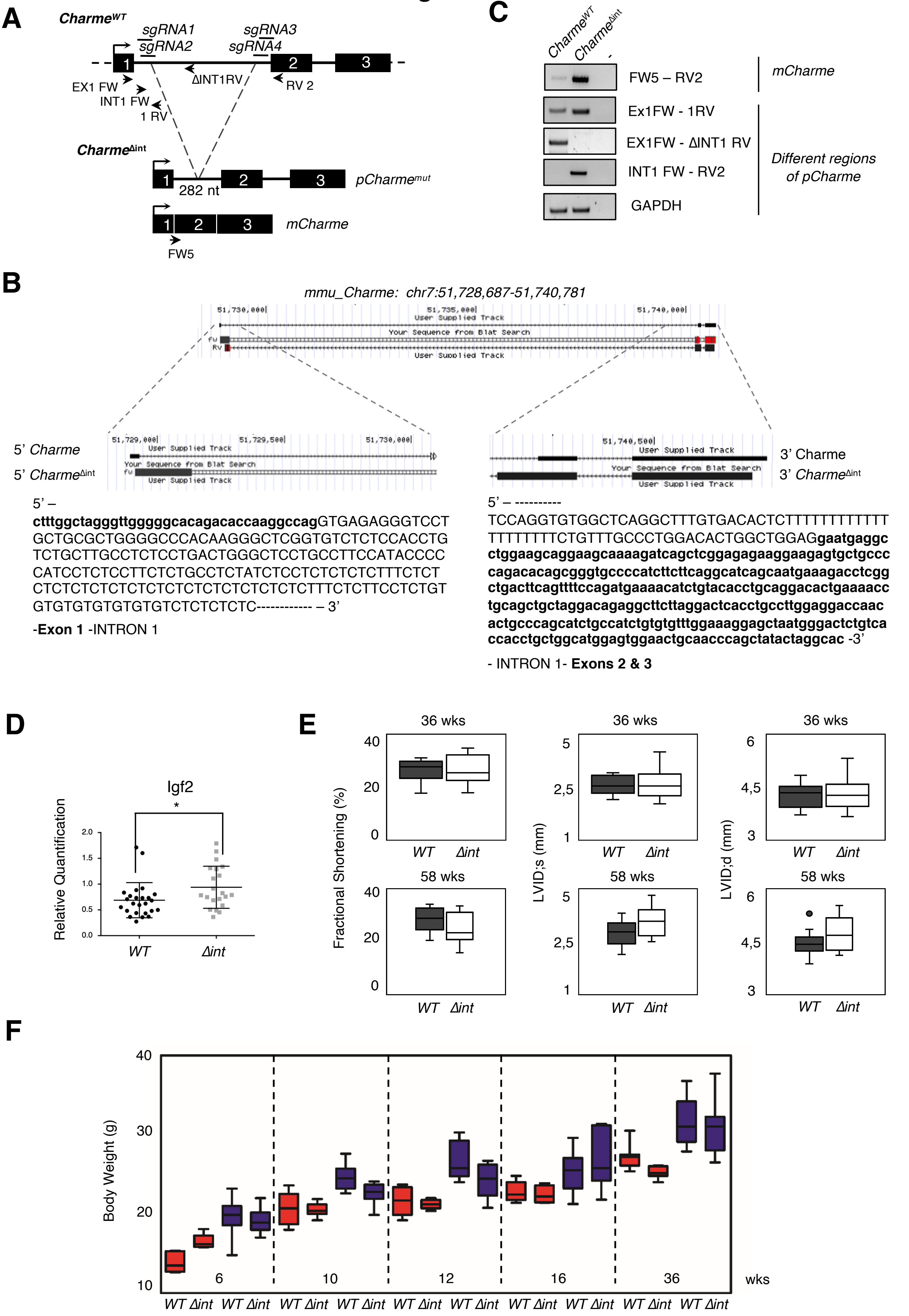
A
CharmeWT
sgRNA1
sgRNA2
sgRNA3
sgRNA4
1
2
3
EX1 FW
INT1 FW
1 RV
ΔINT1RV
RV 2
CharmeΔint
282 nt
pCharmemut
mCharme
FW5
B
mmu_Charme: chr7:51,728,687-51,740,781
5' Charme
5' CharmeΔint
3' Charme
3' CharmeΔint
5' –
ctttggctagggttgggggcacagacaccaaggccagGTGAGAGGGTCCT
GCTGCGCTGGGGCCCACAAGGGCTCGGTGTCTCTCCACCTG
TCTGCTTGCCTCTCCTGACTGGGCTCCTGCCTTCCATACCCC
CATCCTCTCCTTCTCTGCCTCTATCTCCTCTCTCTCTTTCTCT
CTCTCTCTCTCTCTCTCTCTCTCTCTCTCTTTCTCTTCCTCTGT
GTGTGTGTGTGTGTCTCTCTCTC------------ – 3'
-Exon 1 -INTRON 1
5' – ----------
TCCAGGTGTGGCTCAGGCTTTGTGACACTCTTTTTTTTTTTT
TTTTTTTTCTGTTTGCCCTGGACACTGGCTGGAGgaatgaggc
ctggaagcaggaagcaaaagatcagctcggagagaaggaagagtgctgccc
cagacacagcgggtgccccatcttcttcaggcatcagcaatgaaagacctcgg
ctgacttcagttttccagatgaaaacatctgtacacctgcaggacactgaaacc
tgcagctgctaggacagaggcttcttaggactcacctgccttggaggaccaac
actgcccagcatctgccatctgtgtgtttggaaaggagctaatgggactctgtca
ccacctgctggcatggagtggaactgcaacccagctatactaggcac -3'
- INTRON 1- Exons 2 & 3
C
CharmeWT
CharmeΔint
-
FW5 – RV2
mCharme
Ex1FW - 1RV
EX1FW - ΔINT1 RV
INT1 FW - RV2
GAPDH
Different regions of pCharme
D
Igf2
Relative Quantification
WT
Δint
E
36 wks
58 wks
Fractional Shortening (%)
LVID;s (mm)
LVID;d (mm)
WT
Δint
F
Body Weight (g)
6
10
12
16
36
wks
WT Δint WT Δint WT Δint WT Δint WT Δint WT Δint WT Δint WT Δint WT Δint WT Δint

Figure S4. ***In vivo*** **study of *pCharme* intron-1 cardiac function, related to Figure 4**: (**A**) Schematic representation of wild type (*Charme*^{WT}) and *Charme* edited (*Charme*$^{\Delta int}$) genomic loci. The position of the PCR primers and the single guide RNAs (sgRNA) used in this study are shown. The two isoforms (*pCharme*^{mut} and *mCharme*) produced by the edited locus are also shown. See **Table S1** for sgRNAs and primer sequences. (**B**) Screenshot from UCSC genome browser (NCBI37/mm9) to represent the sequences of the *Charme*$^{\Delta int}$ edited locus and the wild type one. DNA sequencing confirmed that in *Charme*$^{\Delta int}$ locus 206 and 76 nucleotides were left at the 5' and 3' ends of intron-1, respectively. Exons are in bold. **(C)** sqRT-PCR quantification of *Charme* gene on *Charme*^{WT} and *Charme*$^{\Delta int}$ heart tissue on 6 weeks old mice. GAPDH mRNA serves as control. -, RT-minus control. PCR products were sequenced and correspond to the expected fragments. See **Table S1** for primer sequences. (**D**) qRT-PCR quantification of Igf2 transcript in *Charme*^{WT} (WT) (black dots) and *Charme*$^{\Delta int}$ (Δint) (grey dots) heart tissue from 2 weeks old mice. Data were normalized to HPRT mRNA. *Charme*^{WT}: n= 25, *Charme*$^{\Delta int}$: n= 21. (**E**) Echocardiographic measurement of left ventricular fractional shortening and internal dimensions at end-systole (LVID;s) and end-diastole (LVID;d) of 36 weeks old (upper panel) and 58 weeks old (lower panel) WT (dark grey box) and Δint (white box) male hearts. 36 weeks old *Charme*^{WT}: n= 9, *Charme*$^{\Delta int}$: n= 13. 58 weeks old *Charme*^{WT} : n= 14 *Charme*$^{\Delta int}$: n= 11.
(**F**) Body weights of female (red boxes) and male (blue boxes) Δint and WT mice from 6 to 36 weeks of age.
Data information: *$P < 0.05$, unpaired Student's t-test.
